# Smoking and vaping alter genes related to mechanisms of SARS-CoV-2 susceptibility and severity: a systematic review and meta-analysis

**DOI:** 10.1183/13993003.00133-2024

**Published:** 2024-07-25

**Authors:** Rachel Bowsher, Timothy H. Marczylo, Karen Gooch, Alexis Bailey, Matthew D. Wright, Emma L. Marczylo

**Affiliations:** 1 Toxicology Department, UK Health Security Agency, Chilton, UK; 2 Pharmacology Section, St George's University of London, London, UK; 3 Vaccine Development and Evaluation Centre, UK Health Security Agency, Salisbury, UK

## Abstract

**Background:**

Evidence for the impact of smoking on coronavirus disease 2019 (COVID-19) is contradictory, and there is little research on vaping. Here we provide greater clarity on mechanisms perturbed by tobacco cigarette, electronic cigarette and nicotine exposures that may impact the risks of infection and/or disease severity.

**Methods:**

Following Preferred Reporting Items for Systematic Reviews and Meta-Analyses (PRISMA) guidelines, the Ovid and Web of Science databases were searched. Study design and exposure-induced gene expression changes were extracted. Each study was quality assessed and higher confidence scores were assigned to genes consistently changed across multiple studies following the same exposure. These genes were used to explore pathways significantly altered following exposure.

**Results:**

125 studies provided data on 480 genes altered by exposure to tobacco cigarettes, e-cigarettes, nicotine or severe acute respiratory syndrome coronavirus 2 (SARS-CoV-2). Genes involved in both SARS-CoV-2 viral-entry and inflammation were changed following exposure. Pathway analysis revealed that many of those genes with high confidence scores are involved in common cellular processes relating to hyperinflammatory immune responses.

**Conclusion:**

Exposure to tobacco cigarettes, e-cigarettes or nicotine may therefore impact initial host–pathogen interactions and disease severity. Smokers and vapers of e-cigarettes with nicotine could potentially be at increased risk of SARS-CoV-2 infection, associated cytokine storm, and acute respiratory distress syndrome. However, further research is required, particularly on e-cigarettes, to determine the biological mechanisms involved in perturbation of viral-entry genes and host–pathogen interactions and subsequent responses within the respiratory tract. This will improve our physiological understanding of the impact of smoking and vaping on COVID-19, informing public health advice and providing improved guidance for management of SARS-CoV-2 and other respiratory viruses.

## Shareable abstract

**Cigarette smoke and electronic cigarettes alter genes and subsequent pathways of interest involved in SARS-CoV-2 entry and hyperinflammatory immune responses. Direct implications on risk of infection and disease severity require more research.** https://bit.ly/4eh060L

## Introduction

The global coronavirus disease 2019 (COVID-19) pandemic and its causative pathogen, severe acute respiratory syndrome coronavirus 2 (SARS-CoV-2), has been responsible for millions of mortalities worldwide [1]. SARS-CoV-2 continues to transmit through the population and poses a threat to public health globally. Host cell entry is predominantly through the angiotensin-converting enzyme (ACE)2 receptor, which is a part of the renin angiotensin system (RAS) [2, 3]. Entry is further enhanced with the priming of the spike proteins of the virus by host cell enzymes transmembrane serine protease (TMPRSS)2, furin, neuropilin (NRP)1 receptor, CD147 receptor and/or cathepsins [4–10]. Following the initial infection, COVID-19 symptoms are flu-like, with severe cases involving a hyperinflammatory response that can result in a cytokine storm and further complications such as acute respiratory distress syndrome (ARDS) and cardiac failure [11]. While vaccines are widely available and act to reduce severe disease, it is important to identify specific at-risk populations to ensure there is targeted public advice.

Within the literature, the epidemiological associations between smoking and COVID-19 appear contradictory. Some studies report that current smokers have a reduced risk of SARS-CoV-2 infection, while others suggest that current smokers have a higher risk of COVID-19 hospitalisation than former or never-smokers [12]. Initial epidemiological investigations using hospital records reported that many smokers required aggressive interventions and ventilation [13], but when adjusting for comorbidities these outcomes became nonsignificant in other studies [14, 15]. This is likely due to the many comorbidities associated with smoking, highlighting the difficulty of distinguishing the impact of smoking alone on COVID-19 and the need for mechanistic studies to underpin the biological plausibility of epidemiological associations. Current mechanistic evidence is largely centred around ACE2, nicotinic acetylcholine receptors (nAChRs) and RAS, with potential crosstalk between ACE2 and nAChRs *via* RAS implicated in both reduction of SARS-CoV-2 infection [12, 16, 17] and more severe COVID-19 through stimulation of inflammatory signalling pathways [18–20]. This suggests complex mechanisms that are dependent on infection/disease stage.

E-cigarette use in the UK is increasing as many current smokers use vaping as a tool to stop smoking. Many ex-smokers continue to vape and the number of never-smokers that have begun vaping is increasing [21]. Despite this, there is little research on the susceptibility of e-cigarette users to COVID-19. Initial indications suggest they may have an increased risk of SARS-CoV-2 infection, but this may differ with nicotine content, flavours and propylene glycol (PG):vegetable glycerine (VG) content [22, 23].

The aims of this review were therefore to 1) identify key genes and pathways of interest altered by tobacco cigarette, e-cigarette or nicotine exposures that may affect viral-entry (and therefore the risk of an individual to SARS-CoV-2 infection) and associated disease severity; and 2) perform a weight-of-evidence based meta-analysis of key mechanistic studies to clarify the existing contradictory literature on the potential impacts of smoking and vaping on COVID-19.

## Materials and methods

### Search strategy

Following the Preferred Reporting Items for Systematic Reviews and Meta-Analyses guidelines, a thorough search of the literature, using Ovid and Web of Science databases, was undertaken (up to November 2022). Within each database, two individual searches were carried out, one related to smoking exposure (tobacco cigarette smoke/tobacco cigarette smoke condensate (herein referred to as cigarette smoke (CS)), vaping (e-liquid/e-liquid condensate/e-cigarette vape (herein referred to as e-cigarette), or nicotine), pathways of interest and the respiratory tract; and a second focused on the interaction of respiratory viruses (with a focus on SARS-CoV-2), smoking exposure and pathways of interest (detailed in supplementary material S1). Additional search terms related to heated tobacco products were considered, but did not provide any further results eligible for inclusion. Results were collated and duplicates removed.

### Eligibility and exclusion criteria

Studies were screened for eligibility by their title, abstract and full text. R. Bowsher screened all studies, with 10% of the total screened by E.L. Marczylo and A. Bailey. Discrepancies were discussed, and a consensus decision was made. Inclusion and exclusion criteria (table 1) were developed to identify key pathways altered following CS, e-cigarette or nicotine exposure in respiratory epithelial cells that may affect the risk of a normal, healthy individual to SARS-Cov-2 infection and/or COVID-19 severity. Thus, we were interested in the impact of smoking/vaping/nicotine on healthy individuals only and not those with other respiratory pathologies or nonviral respiratory infections.

**TABLE 1:** Inclusion and exclusion criteria for selected studies

**Included studies**	Primary literature articles published in English Focused on epithelial cells within the respiratory tract and exposure to cigarette smoke, e-cigarettes, nicotine and/or a respiratory virus
**Excluded studies**	Focused on either epithelial mesenchymal transition, COPD, cancer, pregnancy, cystic fibrosis, pulmonary sarcoidosis or other respiratory cell types such as endothelial cells Used cancer-derived cell lines or samples from patients with comorbidities such as lung cancer or COPD Did not include exposure information Studied bronchial alveolar lavage or immune cells only

### Data extraction

Data from eligible studies were extracted manually and collated (R. Bowsher). Extracted data included details of exposure (type, dose and time, brand), model (species, cell type) and findings (mRNA and protein) (supplementary material S2). Where supplementary information was available, the data for the top 10 upregulated and/or downregulated genes were extracted.

### Quality assessment

A quality-scoring tool was adapted from previous reviews [24, 25]. Included studies were assessed against six domains designed to measure their ability to address the review aim. These were cell model, route of exposure, dose, gene expression, cytotoxicity and SARS-CoV-2 challenge (supplementary material S3). Each study was given a score for each domain; higher scores were assigned for the following:

1) Physiologically relevant models such as primary epithelial air–liquid interface (ALI) cultures *in vitro* and nonhuman primates *in vivo*.
2) Physiologically relevant routes of exposure such as aerosol systems *in vitro* and intranasal administration *in vivo.*
3) Human exposure relevant doses with appropriate controls or dose-dependent responses.
4) Validation of gene expression changes with knockout, silencing, inhibitor or agonist investigations.
5) Use of multiple cytotoxicity assays (oxidative stress, DNA damage, barrier integrity, *etc.*).
6) SARS-CoV-2 challenge with wild-type infection.

Domain scores were combined and averaged to form an overall quality rating of very low, low, medium or high for each study.

### Meta- and pathway-analysis

A weight-of-evidence approach was developed to generate a confidence score (for the mRNA and/or protein) that combined study frequency (the number of studies investigating the gene of interest (GOI)), consistency (the overall level of change of the GOI across all studies) and quality (the overall quality score of the studies from which each GOI was extracted). The frequency of all upregulated GOI extracted from high-quality studies were multiplied by 2, medium quality by 1.5, low quality by 1 and very low quality by 0.5, while the frequency of all downregulated GOI extracted from high-quality studies were multiplied by −2, medium quality by −1.5, low quality by −1 and very low quality by −0.5. Values for each GOI within each exposure type were combined to generate overall confidence scores for the mRNA and protein per GOI per exposure. Note that e-cigarette data were subdivided into those with nicotine (EC+N) or without nicotine (EC−N). The higher the positive confidence score, the more robust the evidence for upregulation and the greater the negative confidence score, the more robust the evidence for downregulation. Ensembl was used to convert any nonhuman GOI into their human homologues prior to pathway analysis.

To simplify further for pathway analysis, mRNA and protein confidence scores for GOI were combined for each exposure. GOI with the 10 greatest upregulated and downregulated confidence scores for each exposure type (where data were available) were imported into Cytoscape (version 3.10.0) for pathway analysis using the Kyoto Encyclopedia of Genes and Genomes database (22 May 2022). Overall scores for pathways identified as significant or as having a large proportion of high-confidence GOI present were generated by combining the confidence scores of all extracted genes for every exposure (where data were available).

## Results

### Overview of search results

**FIGURE 1:**
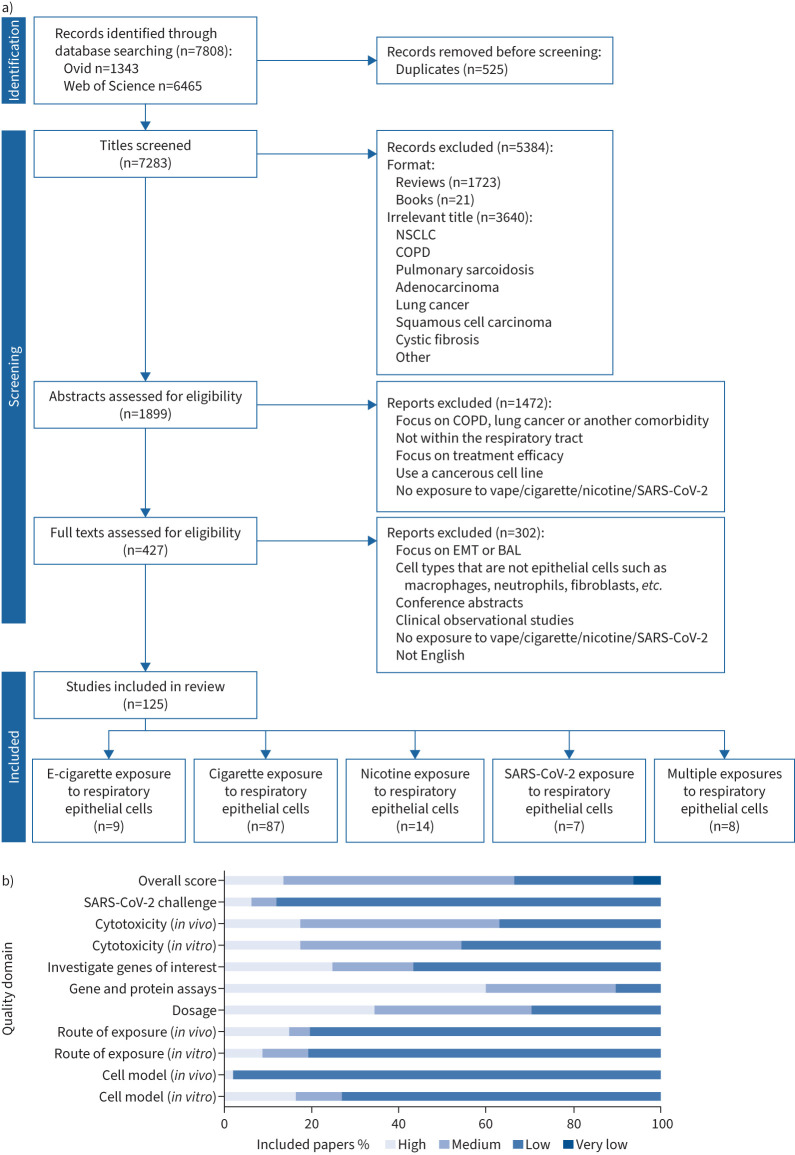
a) Preferred Reporting Items for Systematic Reviews and Meta-Analyses flowchart displaying the search and selection process for studies included in the systematic review. b) Summary of the quality scores assigned to the included studies based on the assessment of domains designed to determine the ability of each study to address the review aims. NSCLC: nonsmall cell lung cancer; SARS-CoV-2: severe acute respiratory syndrome coronavirus 2; EMT: epithelial–mesenchymal transition; BAL: bronchoalveolar lavage; e-cigarette: electronic cigarette.

Of the 7808 records identified by the literature search, 125 were selected for inclusion and categorised by exposure type (figure 1a). Many excluded studies focused on comorbidities rather than investigating the response within a healthy respiratory system. Within the included studies, most investigated the epithelial cell response to CS (n=87, 70%) with fewer on either e-cigarette, nicotine or SARS-CoV-2 exposures (n=9 (7%), n=14 (11%) and n=7 (5%), respectively) or multiple exposure types (n=8, 5%). The majority of included studies were performed *in vitro* (n=78, 62%) *versus in vivo* (n=22, 18%), while 25 (20%) used both *in vitro* and *in vivo* models. Most *in vitro* studies used human epithelial cell lines or primary cells (n=71, 57%), with 31 (25%) using other species (including mouse, rat, ferret, guinea pig, sheep and non-human primates) and 23 (18%) using human plus another species. Throughout the included studies, models, routes of exposure, doses, end-point assays, GOI, cytotoxicity and/or viral challenge varied, making direct comparisons across the included studies difficult.

### Quality assessment

Quality scores per domain per study are detailed in supplementary material S4. The majority of the included studies were high or medium quality overall (67%, n=84), with only 6% (n=8) of very low quality (figure 1b). Of the *in vitro* studies, 28 (27%) used high-quality cell models such as primary cells cultured at ALI for any length of time, or cell lines cultured at ALI for ≥14 days allowing the cells to differentiate into a pseudostratified epithelium. The remaining *in vitro* studies used submerged monolayer cell culture or cell-line ALI cultures for <14 days, which may therefore not be fully differentiated. The route of exposure for *in vitro* studies was predominantly *via* direct application of a solution containing tobacco cigarette condensate, e-liquid or nicotine (n=94, 91%) *versus* aerosolised delivery (n=9). Likewise, almost all *in vivo* studies used a low-quality model (n=46, 98%) combined with a low-quality route of exposure (n=37, 81%). Only 16 (12%) included studies directly measured the effect of CS, e-cigarettes or nicotine on infection using wild-type or pseudo-SARS-CoV-2. However, 90% (n=112) of the studies performed some additional investigation or validation of exposure-induced gene expression changes.

### Meta-analysis

#### Genes of interest

**FIGURE 2:**
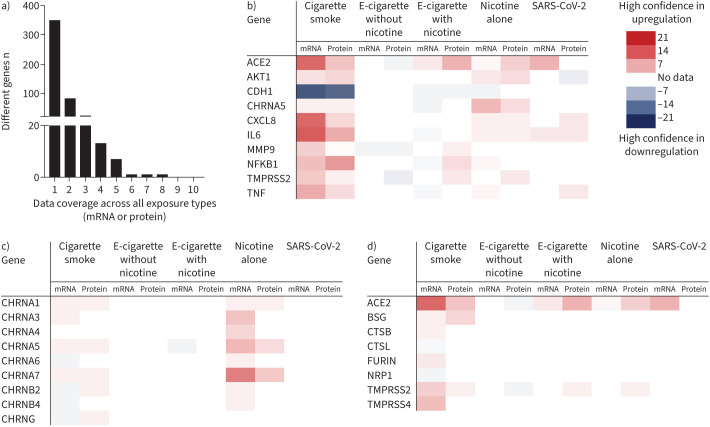
a) The variety of exposure data (mRNA/protein and type) available for each gene; b) the genes with data available for five or more fields (mRNA/protein for each exposure type); c) nicotinic acetylcholine receptor changes following different exposures; and d) those known to have roles in severe acute respiratory syndrome coronavirus 2 (SARS-CoV-2) viral entry. e-cigarette: electronic cigarette; ACE: angiotensin-converting enzyme; NRP: neuropilin; TMPRSS: transmembrane serine protease.

While included studies provided data on exposure-induced expression changes in 480 genes (supplementary material), the vast majority (n=351, 73%) were only measured in one article and were thus assigned a single confidence score (figure 2a). Of those that were assigned two confidence scores (n=82, 17%), many were only measured in one exposure type, predominantly CS. Despite obvious data gaps and variations in confidence across exposures, some exposure-induced trends were identified.

Those genes with five or more confidence scores are shown in figure 2b. These GOI have the most data available to compare across different types of exposures. Many of these genes (ACE2, AKT1, CHRNA5, CXCL8, interleukin (IL)6, matrix metalloproteinase (MMP)9, NFκB1, TMPRSS2, tumour necrosis factor (TNF)) were upregulated by CS. In contrast, e-cigarettes downregulated CDH1, CHRNA5, IL6 and TNF. E-cigarette-induced changes in ACE2, TMPRSS2 and MMP9 were less clear, with some contrasting evidence depending on the presence of nicotine (upregulated with nicotine and downregulated without). Exposure to nicotine alone followed a similar trend to CS with upregulation of ACE2, AKT1, CHRNA5, CXCL8, IL6, NFκB1, TMPRSS2 and TNF, and downregulation of CDH1. EC+N also downregulated CDH1, and exposure to SARS-CoV-2 induced upregulation of ACE2, CXCL8, IL6 and TNF.

Exposure-induced changes in additional nAChRs and SARS-CoV-2 viral-entry genes are shown in figure 2c and d. The data on nAChRs predominantly comes from exposures to nicotine alone, which upregulated CHRNA1–7 and CHRNB2/4. In addition, there was some evidence for CS-induced upregulation of CHRNA1/3/5/7 and downregulation of CHRNA6/B4, with contrasting results for CHRNB2/G. The data on other viral-entry genes comes from exposures to CS, which provided some evidence for upregulation of BSG, CTSB, FURIN and TMPRSS4, and downregulation of CTSL and NRP1.

#### Pathway analysis

**FIGURE 3:**
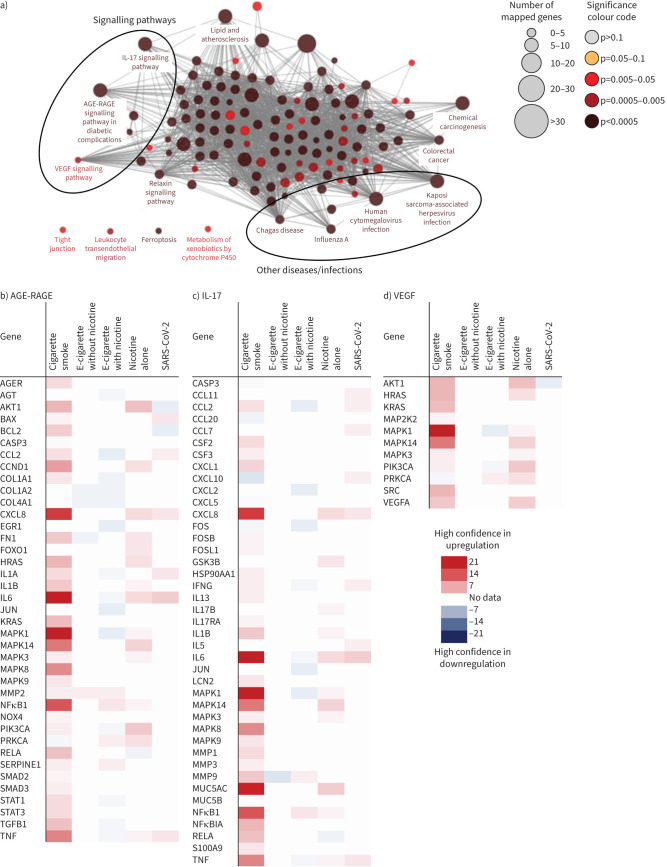
a) Pathway analysis of altered genes following exposure to cigarette smoke, electronic cigarettes (e-cigarettes) and nicotine. b–d) The confidence scores of genes altered following exposure (to cigarette smoke, electronic cigarettes, without or with nicotine and severe acute respiratory syndrome coronavirus 2 (SARS-CoV-2) alone) that are involved in the pathways of interest: b) advanced glycation end-products (AGE)-receptors for advanced glycation end-products (RAGE) (in diabetic complications), c) interleukin (IL)17 and d) vascular endothelial growth factor (VEGF) signalling. MAPK: mitogen-activated protein kinase; TNF: tumour necrosis factor; MMP: matrix metalloproteinase.

Significantly altered pathways following CS, e-cigarette or nicotine exposures included advanced glycation end-products (AGE)-receptors for advanced glycation end-products (RAGE) (in diabetic complications), IL17 and vascular endothelial growth factor (VEGF) signalling, with links to other diseases/infections such as Chagas disease, influenza A, human cytomegalovirus and Karposi sarcoma-associated herpesvirus (figure 3a). Examination of the confidence scores of the gene expression changes behind these pathways (figure 3b–d) demonstrated that CS upregulated many of the genes in the AGE-RAGE (in diabetic complications), IL17 and VEGF signalling pathways. While data for these genes following SARS-CoV-2 and e-cigarette exposures (particularly EC−N) were limited, there was some evidence that, in contrast to CS, EC-N and EC+N downregulated GOI within the same three pathways. Nicotine- or SARS-CoV-2-included GOI changes more closely resembled those following CS exposure.

In summary, while data were limited for the impact of EC−N, genes and pathways of interest identified in this review were altered following exposure to CS, EC+N, or nicotine alone, some of which were similarly altered by SARS-CoV-2 infection.

## Discussion

Data were extracted from 125 studies identifying genes and pathways perturbed by CS, e-cigarette, nicotine alone or SARS-CoV-2 infection to investigate the potential impact of smoking/vaping/nicotine on the risk of SARS-CoV-2 infection and disease severity. This identified potential biological mechanisms for further investigation, but also highlighted knowledge gaps and factors to consider when collating and interpreting evidence.

### Study design

Many studies used either cell donors with comorbidities, or models (*in vivo* and *in vitro*) that scored as low quality because they lacked physiological relevance. More physiologically relevant *in vitro* cell models include microfluidic systems, three-dimensional co-cultures and primary human epithelial cells cultured at an ALI for a suitable length of time, which allows differentiation of basal cells into ciliated or mucus-producing goblet cells with tight junctions that form an epithelial barrier [26, 27]. Cells cultured at ALI have their apical surface in contact with air, enabling aerosol exposures. ALI combined with aerosol exposures have greater physiological relevance compared to submerged cultures (test substance added into the media covering cell monolayer) or suspension exposures (test substance added in solution to ALI culture). Cellular responses following aerosol exposure, including the release of cytokines, are more likely indicative of the aerosol constituents, rather than the result of stress from the abnormal environment within submerged cultures or suspension exposures. Nevertheless, more physiologically relevant models are time-consuming, expensive and can create large data variability, especially when using primary cells from multiple donors. Cell lines differentiated at ALI are an alternative, less variable, option. However, full validation is essential to fully characterise physiological relevance and presence of key mechanisms [26, 27]. Inconsistencies within study models, exposure methods and doses, made comparing overall outcomes challenging.

Extracting mRNA and protein data also revealed inconsistencies. Much of the data available following exposure provided information on either mRNA or protein levels alone, creating gaps in the dataset. There was little weight of evidence as many GOI were only reported in one study. Only ACE2 expression data were available following all exposures. Studies on e-cigarettes were particularly lacking, with the impacts of different PG:VG content, flavours and nicotine composition remaining largely unstudied [21, 28].

### Genes of interest

**FIGURE 4:**
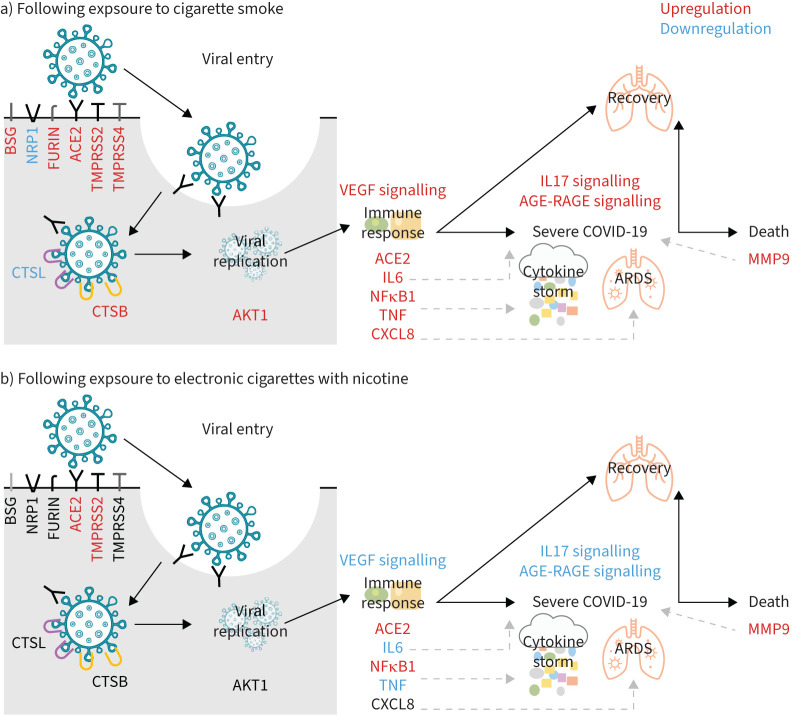
An overview of how genes and signalling pathways of interest may alter the risk of severe acute respiratory syndrome coronavirus 2 infection and subsequent coronavirus disease 2019 (COVID-19) severity following exposure to a) cigarette smoke or b) electronic cigarettes with nicotine. Where text remains black for genes or signalling pathways, there were no data available. NRP: neuropilin; ACE: angiotensin-converting enzyme; TMPRSS: transmembrane serine protease; VEGF: vascular endothelial growth factor; IL: interleukin; TNF: tumour necrosis factor; AGE: advanced glycation end-products; RAGE: receptors for advanced glycation end-products; ARDS: acute respiratory distress syndrome; MMP: matrix metalloproteinase.

GOI were selected as 1) having key roles in SARS-CoV-2 viral-entry (ACE2, TMPRSS2, TMPRSS4, NRP1, BSG, FURIN, CTSL, CTSB); 2) potentially explaining contradictory results/findings (nAChRs and relevant subunits); and/or 3) within the top 10 genes with the greatest coverage across the different exposure types (ACE2, AKT1, CDH1, CHRNA5, CXCL8, IL6, MMP9, NFKB1, TMPRSS2, TNF). Key GOI are discussed here (and summarised in figure 4) with respect to potential impacts on risk of SARS-CoV-2 infection and COVID-19 severity.

#### Risk of infection

The roles of ACE2 and TMPRSS2 in SARS-CoV-2 viral-entry are well reported and TMPRSS4, NRP1, BSG (also known as CD147), FURIN, CTSL and CTSB also assist with viral-entry [29]. ACE2 was upregulated by SARS-CoV-2 infection [30–33] and ACE2 plus many of these other viral-entry GOI were consistently upregulated by CS [23, 30, 32, 34–44], with only three contradictory studies reporting ACE2 [35, 45] and NRP1 [46] downregulation. Significantly increased SARS-CoV-2 or pseudoviral infection rates were also measured in nasal epithelial cells from smokers [30] and primary human bronchial epithelial cells cultured at ALI [47], compared to controls. Hence, smokers may be at greater risk of infection with SARS-CoV-2 than nonsmokers.

Exposure to EC+N generally upregulated ACE2 [23, 48–50] and TMPRSS2 [50]. One study reported downregulation in ACE2 [22], with more evidence of downregulation induced by EC−N [49, 50]. This suggests that nicotine plays a key role in the impact of e-cigarettes on viral uptake, potentially increasing the risk of SARS-CoV-2 infection in vapers *versus* nonvapers.

Nicotine-enhanced viral uptake is suggested to involve nAChRs. The α7 [38, 46, 51, 52] nAChR and α5 [53], α3 [54] and α1 [53] related subunits were upregulated following exposure to CS, with limited data following e-cigarette exposure. Nicotine-induced upregulation of ACE2 was mediated by α7 nAChR in mice [49] and human bronchial epithelial cells grown in monolayer exposed to nicotine at a dose equivalent to smoking one cigarette [55]. Both studies validated findings, using α7 nAChR knockout experiments or gene silencing. The nicotine-derived nitrosamine ketone within tobacco smoke also upregulated α7 nAChR, increasing the sensitivity of small bronchial epithelial cells to stresses [46]. Wider literature suggests that in response to stress, ACE2 levels increase, as part of RAS, to elicit stress-dampening actions [56] with the promotion of oxidative stress in ACE2 knockout mice [57]. Stimulation of α7 nAChR following exposure to nicotine may increase ACE2 as demonstrated in bronchial epithelial cells [38]. CS and e-cigarettes containing nicotine may therefore promote α7 nAChR-mediated upregulation of ACE2, also potentially increasing the risk of SARS-CoV-2 infection in smokers and vapers *versus* nonsmokers/nonvapers.

Some studies also reported altered expression of key viral-entry genes within different regions of the respiratory tract and between different sexes. ACE2 was upregulated in bronchial cells but downregulated in alveolar cells [36], and, while differences in ACE2 expression were not significantly different in smokers across nasal, bronchial and alveolar tissue, nonsmokers had significantly higher ACE2 expression in alveolar compared to their nasal and bronchial regions (p=0.039 and p=0.007, respectively) [45]. In addition, greater expression of ACE2 was observed in the goblet cells of smokers, and club cells of nonsmokers [41]. With respect to sex differences, one study reported an e-cigarette-induced upregulation of ACE2 mRNA expression in males only [48]; and another observed that, despite a greater ACE2 protein abundance in females, only male ACE2 protein abundance was reduced following PG exposure [49]. Androgen signalling may contribute to these differences since increased androgens in smokers were implicated in the increased expression of both TMPRSS2 and ACE2 [40]. Hence, cell types, respiratory tract region, sex and smoking status can all influence ACE2 expression, which may contribute to the conflicted literature surrounding smoking/vaping and risk of infection with SARS-CoV-2.

#### COVID-19 severity

AKT1 is associated with viral replication [58] and knockdown of AKT or silencing/inhibition of P13K/Akt/mTOR pathways inhibits the replication of respiratory infections such as influenza A [59] and Middle East respiratory syndrome coronavirus (MERS-CoV) [58, 60]. Influenza A and MERS-CoV share transmission and genetic similarities, respectively, with SARS-CoV-2 [61], so exposure-induced changes to AKT1 expression could impact SARS-CoV-2 replication. AKT1 was upregulated with high confidence by CS [51, 62–64] or nicotine alone [51, 55, 65]. This may counteract the viral-induced reduction of AKT reported in one study [66], increasing SARS-CoV-2 replication following infection and the subsequent risk of severe disease in smokers.

Many GOI relate to the pro-inflammatory immune response. The pro-inflammatory cytokines IL6 and TNF, chemokine CXCL8 and/or the NFκB1 inflammatory subunit were upregulated following exposure to CS [18, 35, 64, 67–87], nicotine alone [51, 88–90] or SARS-CoV-2 [32, 35, 66, 91]. While inflammation is a key part of the beneficial immune response, hyperinflammation can be detrimental and the drivers of the switch from beneficial to detrimental remain unknown. An elevated IL6 serum concentration is observed in patients with COVID-19 and is strongly associated with adverse clinical outcomes, suggesting it is a predictor of/linked to more severe disease [92, 93]. TNF and NFκB1 are involved in the cytokine storm and a hyperinflammatory state, and increased levels are indicative of severe COVID-19 [93, 94]. CXCL8 elevation is a prognostic marker for those at a high risk of ARDS and of patients at a high risk of experiencing severe COVID-19 [95, 96]. The induction of a pro-inflammatory environment in smokers may therefore contribute to, and exacerbate, a cytokine storm, leading to more severe COVID-19 and ARDS [93]. There was some limited evidence of e-cigarette-induced IL6 and TNF downregulation, and NFκB1 upregulation [22, 49], suggesting that vapers may be at a lower risk of developing severe COVID-19 *versus* smokers, but at increased risk *versus* nonsmokers.

MMP9 is a matrix metallopeptidase elevated in the plasma of patients with severe COVID-19 and correlated with in-hospital deaths [97]. Upregulation of MMP9 by CS [70, 74, 98–101] and EC+N [49] suggests an increased risk of developing severe COVID-19. The downregulation of MMP9 following exposure to EC−N [49] implies no additional risk for those using nicotine-free e-cigarettes and indicates a role for nicotine in MMP9 expression. The latter is supported by α7 nAChR-mediated upregulation of MMP9 [102], highlighting another potential role for nicotine and nAChRs in COVID-19 severity in addition to increased infection risk.

ACE2 is also part of RAS, which despite originally being identified as the pathway regulating blood pressure, has more recently been shown to play a key role in inflammation [2, 56]. Within RAS, ACE2 and its homologue ACE balance anti- and pro-inflammatory responses, respectively [103]. It is widely reported that the process of SARS-CoV-2 uptake ultimately downregulates ACE2 expression [2]. Thus, while the different roles of ACE2 as the key viral uptake receptor (increasing risk of infection) and mediator of anti-inflammatory responses (protecting against disease) appear contradictory, they should not be considered distinct. Following an initial increase in ACE2-mediated viral uptake, the levels of ACE2 fall, tipping the balance towards ACE and a more pro-inflammatory environment [103]. Thus, the potential impact of smoking/vaping on severe disease through modification of ACE2 is complex and depends on the specific part of the disease process being measured. This is probably a major contributor to the contradictory literature.

Gender differences in SARS-CoV-2 infection risk may also impact COVID-19 severity. In the wider literature, males are frequently reported as having higher rates of COVID-19 mortality and severe disease compared to females [104, 105]. The most plausible explanation for this is gender disparity in hormone levels and immune responses. Oestrogen in females is considered to help modulate the immune system and provide additional protection from severe inflammation [104–106], whereas androgens in males are associated with overactive immune cells and exacerbation of inflammation and disease severity [106]. The latter, in combination with an elevated inflammatory response following exposure to CS or EC+N, may lead to more severe COVID-19 in male smokers.

### Pathways of interest

Pathways of interest were selected as 1) the most significantly enriched (IL17 signalling and AGE-RAGE signalling pathway in diabetic complications) or 2) significantly enriched with wider literature supporting a potential role (VEGF signalling). Key pathways are discussed here (and summarised in figure 4) with respect to potential impacts on risk of SARS-CoV-2 infection and COVID-19 severity.

#### Risk of infection

VEGF signalling drives angiogenesis by inducing cell survival, proliferation and endothelial migration. Most genes involved in VEGF signalling were upregulated following exposure to either CS or nicotine alone [46, 52, 70], including its activator VEGFA [70]. VEGFA is able to activate the VEGF signalling cascade by binding to VEGF [107, 108]. VEGFA also shares a common binding pocket (b12b domain) on the viral-entry receptor NRP1 [10, 109] and therefore may alter SARS-CoV-2 uptake. Upregulation of VEGFA following CS or nicotine exposure [70] could compete with the SARS-CoV-2 spike protein for the NRP1 binding pocket. This highlights the complexity of viral uptake and the need to understand the affinity of SARS-CoV-2 for, and expression levels of, different receptors. While smokers may have less risk of viral-entry *via* NRP1 compared to nonsmokers, SARS-CoV-2 would still be able to enter cells *via* other genes and proteases (such as TMPRSS2 and ACE2), that were upregulated by CS, EC+N or nicotine alone.

#### COVID-19 severity

VEGF signalling may also impact COVID-19 severity. SARS-CoV-2 binding to NRP1 can block VEGF-related signalling, which reduces pain perception [109]. Increased VEGFA is associated with inflammatory-related chronic pain in a variety of conditions [110, 111] and substantially lower levels of VEGFA are reported in the sera of asymptomatic compared to symptomatic COVID-19 patients [112]. Thus, smoking or nicotine induced upregulation of VEGFA and VEGF signalling may lead to greater symptomatic disease.

AGE-RAGE signalling can disrupt the extracellular matrix, enhancing oxidative stress and stimulating NFκB signalling [113]. NFκB1 is a signalling molecule within the AGE-RAGE pathway and, as described earlier, increased levels can be used as a prognostic indicator of severe COVID-19 [94]. AGE-RAGE has been widely studied and implicated in diabetic complications [114] and hyperactive AGE-RAGE signalling in such comorbidities is already considered a risk factor for severe COVID-19 [115, 116]. Exposure to CS or nicotine upregulated many genes within AGE-RAGE signalling with high confidence, including CCND1, CXCL8, HRAS, IL6, KRAS and TNF [18, 22, 32, 64–76, 78–81, 83, 89, 90, 117–121], suggesting that smokers have a hyperactive AGE-RAGE and are therefore more at risk of severe COVID-19. In contrast, the limited data available suggest that EC+N and EC−N do not upregulate the AGE-RAGE pathway and probably do not confer increased disease severity *via* this pathway.

IL17 signalling, encompassing all isoforms, is a pro-inflammatory response attracting chemokines and activating cascades to recruit immune cells to sites of inflammation [122]. Exposure to SARS-CoV-2 increased many genes within the IL17 signalling pathway [32, 66, 91]. Increases in IL17 are observed in COVID-19 patients and associated with the cytokine storm and ARDS, with IL17 blockers being investigated as potential treatments in patients with severe COVID-19 [123]. Similarly, exposure to CS or nicotine upregulated most of the IL17 signalling pathway, including CXCL8, mitogen-activated protein kinase (MAPK)1 and MUC5AC [18, 22, 53, 67, 69–71, 74, 78–83, 85, 90, 100, 124–137]. Elevation of IL17 signalling with further exacerbation following SARS-CoV-2 infection could suggest that smokers may experience more severe disease, whereas the limited data available suggest that EC+N and EC−N do not upregulate IL17 signalling and so are also unlikely to confer increased disease severity *via* this pathway.

It is worth noting that there was overlap in the GOI within, and thus potential cross-talk between, the VEGF, AGE-RAGE and IL17 pathways. This highlights both the complexity of cellular responses to CS, e-cigarettes or nicotine and the need to further investigate and validate specific mechanisms in human-relevant models with appropriate controls and gene/pathway activators/inhibitors.

### Impact of smoking/vaping on the risk/severity of SARS-CoV-2 infection

Overall, the mechanistic evidence to date suggests that cigarette smokers may be at a higher risk of both infection and more severe disease, supporting recently published literature reviews assessing patient outcomes and the potential impact of smoking on such outcomes [138, 139]. While the data on e-cigarettes are limited, there is evidence for a potential increased risk of infection and/or disease severity in vapers of EC+N, with vapers possibly at a lower risk of developing severe COVID-19 *versus* smokers, but at increased risk *versus* nonsmokers. This highlights a key role for nicotine-mediated mechanisms in the health impacts of smoking and vaping.

### Other infections/diseases

Pathway analysis also identified other disease- and infection-related pathways, including human cytomegalovirus, influenza A, Kaposi sarcoma-associated herpesvirus infection and Chagas disease. Both human cytomegalovirus and influenza A are more prevalent in smokers [140–143], providing further support that the results of this review are applicable to wider respiratory infections. In contrast, CS appears to have an inverse relationship with Kaposi sarcoma-associated herpesvirus infection and cancer development [144–146], probably due to virus- and/or disease-specific mechanisms. Chagas disease is a parasitic vector-borne disease that causes immunoinflammatory-driven fibrosis, particularly in the myocardium and digestive system [147], where smoking has been speculated as an underlying risk factor for aspects of severe disease [148]. This highlights the robustness of this review and the methods used; further demonstrates the complexity and variety of downstream responses to smoking; and highlights the importance of investigating smoking- and vaping-related impacts on other communicable diseases.

### Recommendations for future work

The key challenges and knowledge gaps highlighted by this review include study design, lack of studies on e-cigarettes, building on existing literature (including additional cigarette constituents, cell types and/or genes and pathways of interest), risk of infection *versus* disease severity, and application to other infections/diseases. Therefore, we recommend that future work should consider the following.

#### Study design

Study design should address the specific research question within the most physiologically relevant and exposure-relevant model where possible. Models should be 1) selected according to airway region, cell types, sex differences, normal *versus* disease and expression of genes or pathways of interest; and 2) fully characterised and validated. Resulting publications should clearly state the justification for the specific model, exposure route, dose(s) administered and end-points profiled to aid comparison across different studies. It is also important to include studies on normal/healthy models since these are essential to understanding mechanisms before targeting specific populations.

#### E-cigarettes

More research on the cellular responses to e-cigarettes is needed, particularly on genes and pathways of interest highlighted in this review where data were unavailable (AKT1, CDH1, CHRNA5, CXCL8, IL6, NFκB1 and TNF). These studies must compare EC+N and EC−N to further elucidate the role of nicotine in the health impacts of smoking *versus* vaping.

#### Building on existing literature

Development of a list of “core genes/pathways” to further investigate with specific hypothesis-driven studies. This would add to the weight of existing evidence, enable better comparison between studies and could evolve with the expanding literature. The safety of new products, such as e-cigarettes with different compositions or flavours, could then be more rapidly compared to existing products with known impacts. This is particularly pertinent following recent evidence that other constituents of e-cigarette aerosols can impact susceptibility to SARS-CoV-2 infection [149]. Therefore, the literature should be continually reviewed to identify additional cigarette ingredients/compositions (*e.g.* benzoic acid), cell types (*e.g.* endothelial and immune cells), genes and/or pathways (*e.g.* oxidative stress and antioxidant mechanisms) of interest as the evidence grows.

#### Risk of infection versus disease severity

Better understanding of how the processes of infection and subsequent disease development inter-relate and are impacted by smoking/vaping and wider environmental exposures.

#### Other infections/diseases

Similar reviews, incorporating a weight-of-evidence based approach that considers the frequency, consistency and quality of existing literature, should be performed to assess the impact of smoking and vaping on wider infections and diseases with inflammatory mechanisms.

### Conclusions

To our knowledge, this is the first review to assess mechanistic associations between smoking or vaping and SARS-CoV-2 infection and disease severity. Using a novel weight-of-evidence meta-analysis, we have identified genes and pathways of interest within the respiratory tract altered by smoking, vaping and/or nicotine that may impact SARS-CoV-2 infection and/or resulting COVID-19 severity. This suggests that cigarette smokers may be at a higher risk of both infection and more severe disease. Large knowledge gaps remain on the impact of e-cigarettes, with the limited data suggesting a potential increased risk of infection and/or disease severity in vapers of e-cigarettes, particularly those containing nicotine. This highlights a key role for nicotine-mediated mechanisms in the health impacts of smoking and vaping. Further specific hypothesis-driven experimental investigations within more physiologically relevant models and improved study design reporting are required to build on our existing knowledge and promote comparisons across studies. Such work is essential for developing improved public health guidance on the risk of communicable disease infection and severity for potentially more vulnerable populations such as smokers and vapers.

## Supplementary material

10.1183/13993003.00133-2024.Supp1 **Please note:** supplementary material is not edited by the Editorial Office, and is uploaded as it has been supplied by the author.
Supplementary material erj-00133-2024.supplement

## Shareable PDF

10.1183/13993003.00133-2024.Shareable1 This one-page PDF can be shared freely online.
Shareable PDF ERJ-00133-2024.Shareable
