# Supplementary material for: Smoking and vaping alter genes related to mechanisms of SARS-CoV-2 susceptibility and severity: a systematic review and meta-analysis
Source: Eur Respir J. 2024 Jul 25;64(1):2400133. doi: 10.1183/13993003.00133-2024 (PMC11269771; doi:10.1183/13993003.00133-2024)
Supplement: Supplementary file 1 [file erj-00133-2024.supplement.pdf]

Supplementary Information for:

Smoking and vaping alter genes related to mechanisms of SARS-CoV-2 susceptibility and severity

Rachel Bowsher[1,2], Timothy H Marczylo[1], Karen Gooch[3], Alexis Bailey[2], Matthew Darren Wright[1] and Emma Marczylo[1].

[1]Toxicology Department, UK Health Security Agency, Chilton, UK
[2]St George's University of London, London, UK
[3]Vaccine Development and Evaluation Centre, UK Health Security Agency, Salisbury, UK

Contents	Page
Supplementary information 1 – Search string	1
Supplementary information 2 – Data extraction summary	3
Supplementary information 3 – Quality scoring tool	30
Supplementary information 4 – Quality assessment summary	31
Supplementary information 5 – Confidence scoring of genes	38
References	50

Supplementary information 1: Search string for the literature review in OVID and Web of Science (WoS) databases. Search A included exposure, pathways and tissue/region of interest; Search B focused on exposure, pathways and respiratory viruses. Results for search A and B within each database were combined up to November 2022. Note due to the large volume of literature results, any key recent studies since November 2022 have been incorporated into the discussion.

Database and search	Joining term	Subject headers **OR** key words (OVID); Key words (WoS)	Total no. results
OVID search A	-	(exp cigarette smoking/ or exp smoking device/ or exp smoking/ or exp cigar smoking/ or exp vaping associated lung injury/ or exp vaping/) **OR** ((smok*/ or vap*/ or cigarette*/ or e-cigarette*/ or electronic-cigarette*/ or e-cig*/ or nicotine end*/ or electronic cigarette*) or (nicotine/ or tobacco/ or ECIG/ or TCIG/ or ENDS) or ("electronic nicotine delivery system") or (cigarette* adj3 smoke*))	1341
	AND	(exp virus entry/ or exp cholinergic system/ or exp renin angiotensin aldosterone system/ or exp "angiotensin[1-7]"/ or exp angiotensin converting enzyme 2/ or exp angiotensin/ or exp epithelial mesenchymal transition) **OR** ((ACE2/ or RAAS/ or ACE/ or TMPRSS2/ or TMPRSS4/ or ADAM17/ or CHRNA7/ or RAS/ or ANG-II/ or ANG-I/ or Furin/ or Cathepsin/ or hACE2/ or MasR/ or NRP1/ or TFRC/ or AXL/ or ASGR1/ or KREMEN1/ or CD147/ or TfR/ or IFN-beta1/ or IFNB1/ or CTSL/ or CTSB/ or AGTR2/ or AT2/ or AGTR1/ or AT1/ or MAS1/ or nAChRs/ or EMT/ or LZTFL1/ or SNAI*/ or ZEB2/ or IFI27) or ("angiotensin converting enzyme 2"/ or "renin angiotensin aldosterone system"/ or "renin-angiotensin-aldosterone system"/ or "angiotensin converting enzyme"/ or "transmembrane serine protease 2"/ or "transmembrane serine protease 4"/ or "ADAM metallopeptidase Domain 17"/ or "Nicotinic acid receptor"/ or "cholinergic receptor nicotinic alpha 7 subunit"/ or "renin angiotensin system"/ or "angiotensin II"/ or "angiotensin I"/ or "Salicylic acid receptors"/ or "angiotensin receptor"/ or "Neuropilin 1"/ or "transferrin receptor"/ or "cysteine proteases"/ or "angiotensin II receptor type 1"/ or "angiotensin II receptor type 1"/ or "nicotinic acetylcholine receptors"/ or "alpha7 nAChRs"/ or "epithelial-mesenchymal transition"/ or "epithelial mesenchymal transition"/ or "receptor tyrosine kinase"/ or "snail family transcription repressor"/ or "Zinc finger E-box binding homeobox 2"))	
	AND	(exp respiratory system/) **OR** ((bronch*/ or alveol*/ or lung/ or nasal/) or ("respiratory tract"))	
OVID search B	-	(exp cigarette smoking/ or exp smoking device/ or exp smoking/ or exp cigar smoking/ or exp vaping associated lung injury/ or exp vaping/) **OR** ((smok*/ or vap*/ or cigarette*/ or e-cigarette*/ or electronic-cigarette*/ or e-cig*/ or nicotine end*/ or electronic cigarette*) or (nicotine/ or tobacco/ or ECIG/ or TCIG/ or ENDS) or ("electronic nicotine delivery system") or (cigarette* adj3 smoke*))	
	AND	(exp virus entry/ or exp cholinergic system/ or exp renin angiotensin aldosterone system/ or exp "angiotensin[1-7]"/ or exp angiotensin converting enzyme 2/ or exp angiotensin/ or exp epithelial mesenchymal transition) **OR** ((ACE2/ or RAAS/ or ACE/ or TMPRSS2/ or TMPRSS4/ or ADAM17/ or CHRNA7/ or RAS/ or ANG-II/ or ANG-I/ or Furin/ or Cathepsin/ or hACE2/ or MasR/ or NRP1/ or TFRC/ or AXL/ or ASGR1/ or KREMEN1/ or CD147/ or TfR/ or IFN-beta1/ or IFNB1/ or CTSL/ or CTSB/ or AGTR2/ or AT2/ or AGTR1/ or AT1/ or MAS1/ or nAChRs/ or EMT/ or LZTFL1/ or SNAI*/ or ZEB2/ or IFI27) or ("angiotensin converting enzyme 2"/ or "renin angiotensin aldosterone system"/ or "renin-angiotensin-aldosterone system"/ or "angiotensin converting enzyme"/ or "transmembrane serine protease 2"/ or "transmembrane serine protease 4"/ or "ADAM metallopeptidase Domain 17"/ or "Nicotinic acid receptor"/ or "cholinergic receptor nicotinic alpha 7 subunit"/ or "renin angiotensin system"/ or "angiotensin II"/ or "angiotensin I"/ or "Salicylic acid receptors"/ or "angiotensin receptor"/ or "Neuropilin 1"/ or "transferrin receptor"/ or "cysteine proteases"/ or "angiotensin II receptor type 1"/ or "angiotensin II receptor type 1"/ or "nicotinic acetylcholine receptors"/ or "alpha7 nAChRs"/ or "epithelial-mesenchymal transition"/ or "epithelial mesenchymal transition"/ or "receptor tyrosine kinase"/ or "snail family transcription repressor"/ or "Zinc finger E-box binding homeobox 2"))	
	AND	(exp respiratory tract infection/ or exp respiratory virus/) **OR** (COVID-19/ or coronavirus*/ or coronovirus*/ or SARS-CoV-2/ or SARS-CoV-1/ or influenza/ or SARS/ or 2019-nCoV/ or HCoV-19/ or MERS/ or RSV/ or SARSCoV1/ or SARSCoV2/ or SARSCoV19/ or H1N1/ or ("novel coronavirus"))	

S1 continued…

Database and search	Joining term	Subject headers **OR** key words (OVID); Key words (WoS)	Total no. results
WoS search A	-	(smok* or vap* or cigarette* or e-cigarette* or electronic-cigarette* or e-cig* or nicotine end* or electronic cigarette*) or (nicotine or tobacco or ECIG or TCIG or ENDS) or ("electronic nicotine delivery system" or "cigarette smoking" or "smoking device" or "cigar smoking" or "vaping associated lung injury" or "vaping") or (cigarette* NEAR/3 smoke*)	6465
	AND	(ACE2 or RAAS or ACE or TMPRSS2 or TMPRSS4 or ADAM17 or CHRNA7 or RAS or ANG-II or ANG-I or Furin or Cathepsin or hACE2 or MasR or NRP1 or TFRC or AXL or ASGR1 or KREMEN1 or CD147 or TfR or IFN-beta1 or IFNB1 or CTSL or CTSB or AGTR2 or AT2 or AGTR1 or AT1 or MAS1 or nAChRs or EMT or LZTFL1 or SNAI* or ZEB2 or IFI27 or angiotensin) or ("angiotensin converting enzyme 2" or "renin angiotensin aldosterone system" or "renin-angiotensin-aldosterone system" or "angiotensin converting enzyme" or "transmembrane serine protease 2" or "transmembrane serine protease 4" or "ADAM metallopeptidase Domain 17" or "Nicotinic acid receptor" or "cholinergic receptor nicotinic alpha 7 subunit" or "renin angiotensin system" or "angiotensin II" or "angiotensin I" or "Salicylic acid receptors" or "angiotensin receptor" or "Neuropilin 1" or "transferrin receptor" or "cysteine proteases" or "angiotensin II receptor type 1" or "angiotensin II receptor type 1" or "nicotinic acetylcholine receptors" or "alpha7 nAChRs" or "epithelial-mesenchymal transition" or "epithelial mesenchymal transition" or "receptor tyrosine kinase" or "snail family transcription repressor" or "Zinc finger E-box binding homeobox 2" or "virus entry" or "cholinergic system" or "angiotensin[1-7]")	
	AND	(bronch*/ or alveol*/ or lung/ or nasal/) or ("respiratory tract" and "respiratory system").	
WoS search B	-	(smok* or vap* or cigarette* or e-cigarette* or electronic-cigarette* or e-cig* or nicotine end* or electronic cigarette*) or (nicotine or tobacco or ECIG or TCIG or ENDS) or ("electronic nicotine delivery system" or "cigarette smoking" or "smoking device" or "cigar smoking" or "vaping associated lung injury" or "vaping") or (cigarette* NEAR/3 smoke*)	
	AND	(ACE2 or RAAS or ACE or TMPRSS2 or TMPRSS4 or ADAM17 or CHRNA7 or RAS or ANG-II or ANG-I or Furin or Cathepsin or hACE2 or MasR or NRP1 or TFRC or AXL or ASGR1 or KREMEN1 or CD147 or TfR or IFN-beta1 or IFNB1 or CTSL or CTSB or AGTR2 or AT2 or AGTR1 or AT1 or MAS1 or nAChRs or EMT or LZTFL1 or SNAI* or ZEB2 or IFI27 or angiotensin) or ("angiotensin converting enzyme 2" or "renin angiotensin aldosterone system" or "renin-angiotensin-aldosterone system" or "angiotensin converting enzyme" or "transmembrane serine protease 2" or "transmembrane serine protease 4" or "ADAM metallopeptidase Domain 17" or "Nicotinic acid receptor" or "cholinergic receptor nicotinic alpha 7 subunit" or "renin angiotensin system" or "angiotensin II" or "angiotensin I" or "Salicylic acid receptors" or "angiotensin receptor" or "Neuropilin 1" or "transferrin receptor" or "cysteine proteases" or "angiotensin II receptor type 1" or "angiotensin II receptor type 1" or "nicotinic acetylcholine receptors" or "alpha7 nAChRs" or "epithelial-mesenchymal transition" or "epithelial mesenchymal transition" or "receptor tyrosine kinase" or "snail family transcription repressor" or "Zinc finger E-box binding homeobox 2" or "virus entry" or "cholinergic system" or "angiotensin[1-7]")	
	AND	(COVID-19 or coronavirus* or coronavirus* or SARS-CoV-2 or SARS-CoV-1 or influenza or SARS or 2019-nCoV or HCoV-19 or MERS or RSV or SARSCoV1 or SARSCoV2 or SARSCoV19 or H1N1) or ("novel coronavirus" or "respiratory tract infection" or "respiratory virus")	

Supplementary information 2: Data extraction summary of the studies selected for inclusion within the literature review.

Reference	Exposure			Model		Findings	
	Type	Dose and time	Brand	Species	Cell type	Gene (mRNA)	Protein
Agraval, H., et al., 2022	Cigarette smoke extract	100% CSE (2 x cigarettes in 10mls pre-warmed media) diluted to a variety of concentrations for use (0.5-20%)	Marlboro red cigarettes	Human	Primary small airway epithelial cells	Upregulated following CS exposure: COX-2, MMP2, MMP9	
Albano, G.D., et al., 2018	Cigarette	10 to 20% CSE for 3 cycles per day for 4 days.	Commercially available cigarettes (Malboro Red Label, Phillip Morris International, Switzerland)	Cell line	16HBE		Upregulated following CS exposure: PEBP1, BETA2AR, ERK1/2, MAPK1/2, ERK1/2, MACHRM3, CHAT, ACH, IL8, NOX4
Aliee, H., et al., 2020	SARS-CoV-2 and smoking	Patient smoker or non-smoker	N/A	Human	Nasal brush or curettage and bronchial brush or biopsy	Upregulated following CS exposure: ACE2, TMPRSS2, FURIN, BSG. Downregulated following CS exposure: CTSL.	
Al-Wadei, H.A.N., et al., 2010	NNK tobacco component	1ml for 7 days		Cell line	HPL1D (small airway epithelial)	Upregulated following CS exposure: VEGF. Downregulated following CS exposure: NRP1.	Upregulated following CS exposure: VEGF, CHRNA7.
Andrault, P., et al., 2019	Cigarette condensate	pBECs treated with CSE(0-50%) for 2hrs and 24hrs in 5mL media	3R4F	Human	Primary bronchial epithelial cells	Upregulated following CS exposure: P38, MAPK, CPLA2, CYP1A1	Upregulated following CS exposure: CatS, TOS, TAS
Arredondo, J., et al., 2006	NNK or NNN tobacco component	1uM NNK for 24hrs	N/A	Cell line	BEP2D bronchial cells	Upregulated following exposure to CS: PCNA, BCL-2, NF-KB, GATA-3, STAT1.	

S2 continued…

Reference	Exposure			Model		Findings	
	Type	Dose and time	Brand	Species	Cell type	Gene (mRNA)	Protein
Basil, M. et al., 2022.	Cigarette smoke	60mins twice/d for 6 months.	1R6F research cigarettes	Human and Ferret	AT2 cells; Lung.	Upregulated following CS exposure: SCGB3A2, LAMP3	
Blank, U., et al., 1997	Nicotine	0.5 - 3.0mM nicotine	N/A	Human	Nasal epithelial cells	Upregulated following nicotine exposure: Ca2+.	
Cai, G., et al., 2020	Smoking	Non-smoker or smoker	N/A	Human	Lung airway epithelium	Upregulated following CS exposure: ACE2, FURIN	
Carlier, F. et al., 2021	Smoker	Smoker vs non-smoker samples		Human	Primary human bronchial epithelial cells	Upregulated following CS exposure: SPDEF, FOXA3, IL-8, CXCL-8. Downregulated following CS exposure: MYB, FOXJ1, DNAI1	Downregulated following CS exposure: occludin
Carlisle, D.L., et al., 2004	Cigarette smoke and nicotine	Never smoker, ex-smoker and active smoker	N/A	Cell line and Human	Bronchial epithelial cells and BEAS2B cell line	Upregulated following CS exposure: CHRNA1, CHRNA5. Upregulated following nicotine exposure: CA2+, PKC, P38. Downregulated following CS exposure: CHRNB4, CHRNA7, CHRNB2, CHRNAGAMMA.	Upregulated following CS exposure: CHRNA1, CHRNA5, CHRNB2, CHRNAGAMMA.
Chakladar, J., et al., 2020	Smoking	Smokers vs former smokers	N/A	Human	Lung and oral epithelium	Upregulated following CS exposure: ACE2, TMPRSS2, ADAM17, ANDROGEN RECEPTOR	
Chen, P., et al., 2021.	Smoking	CSE 1-4% for 24h. 100% CSE is 10 cigarettes bubbled into 25ml media.	Suyan, Jiangsu Cigarettes, China	Cell line and human	Lung tissue and 16HBE cells	Upregulated following CS exposure: EGR3, BAX, CASPASE-3, BCL-2, IL6, TNFALPHA, COX2, XIST	

S2 continued…

Reference	Exposure			Model		Findings	
	Type	Dose and time	Brand	Species	Cell type	Gene (mRNA)	Protein
Chen, Q., et al., 2021	SARS-CoV-2			Mice	Humanised ACE2 mice via CRISPR/Cas9	Upregulated following SARS-CoV-2 exposure: IL-1alpha, IL-2, IL-5, IL-6, IL-17A, IL28, IL-22, IP-10, MCP-1, MCP-3, Eotaxin, IFNgamma.	
Chen, Q., et al., 2022	SARS-CoV-2	Victorian Infectious Diseases Reference strain at an MOI of 0.05, 0.1 or 1 for 1h at 37 degrees Celsius		Human	Primary human bronchial epithelial cells; Bci and BEAS2b cell line		Upregulated following SARS-CoV-2 exposure: ZO-1
Chu, M., et al., 2005	Nicotine	0.5uM nicotine supplemented into media for a constant concentration and exposure for 8 wks	N/A	Mouse	Lung epithelial cells (LA4)		Upregulated following nicotine exposure: Ras, EGFR, Raf, P13K, cyclin D1.
Chung, S., et al., 2015	Smoking	60mg/m^3 6h/d for 5 days	3R4F Kentucky	Human and Mice	Airway epithelial cells and 129/Sv mice	Upregulated following CS exposure: nSMase2, SRC, MAPK, P38.	
Chung, S., et al., 2019	E-cig with or without nicotine and nicotine alone	Vapour containing 36mg/ml nicotine for 24h or 1uM nicotine	Joytech VTC mini (SciReq, Montreal Canada)	Human and sheep	Primary bronchial epithelial cells and tracheal secretions	Upregulated following nicotine exposure: TRAP1	
Cortijo, J., et al., 2011	Cigarette and nicotine	Up to 30% CSE (10%, for 24hr). Direct exposure to nicotine (10uM)	-	Human	Bronchial lung tissue	Upregulated following CS exposure: MUC5AC, EGFR, P42/P44, M3	Upregulated following CS exposure: MUC5AC, M3
Daniell, H., et al., 2022	SARS-CoV-2	Infected patients		Human	Lung autopsy		

S2 continued…

Reference	Exposure			Model		Findings	
	Type	Dose and time	Brand	Species	Cell type	Gene (mRNA)	Protein
Di Vincenzo, S. et al., 2022	Whole cigarette smoke	4-5 mins CS exposure with 10 min ventilation	3R4F Cigarettes	Human	Primary bronchial epithelial cells	Upregulated following CS exposure: SNAIL1, ZEB1, SNAIL2. Downregulated following CS exposure: CDH1, VIMENTIN, NOTCH1, E-CADHERIN, MMP9, TP63, SCGB1A1, JAG1, HES1.	Downregulated following CS exposure: E-CADHERIN, VIMENTIN.
Downs, C., et al., 2011	Cigarette	1, 10, 50, 100% CSE for 24hrs		Rat	Alveolar epithelia type 1 cells	Upregulated following CS exposure: ERCC6, ZMYND17, NQO1. Downregulated following CS exposure: APOE, CYGB, PRNP, SLC38A1, TMOD1	
Du, H., et al., 2012	Cigarette	10 mins every other day (1,5 or 20% smoke concentration)	Xuzhou cigarette factory, China (contain 11mg tar oil and 0.8mg nicotine)	Cell line	BEAS-2B	Upregulated following CS exposure: Survivin. Downregulated following CS exposure: MGMT, RASSF1A.	Downregulated following CS exposure: Caspase-3
Ebrahimpour, A., et al., 2019	Nicotine	1nM, 10nM and 100nM for 24h	N/A	Human	Primary epithelial cells	Upregulated following nicotine exposure: TNFRSF1B, TNFRSF4, TNF-ALPHA, NFKB, ILIBETAIL2RB, IL17B, IL21 RECEPTOR, TWIST1, CHRNA7, ALPHA7 NACHR	
Eurlings, I., et al., 2014	Cigarette	BEAS-2B 1% CSE for 15 mins twice every 24hrs. Mice were exposed 4 times per day, 5 days a week for 24 weeks	Filters were removed from 3R4F Research cigarettes	Cell line and mice	BEAS-2B	Upregulated following CS exposure: PAL1, vimentin, collagen, CA9, HIf-1ALPHA. Downregulated following CS exposure: E-cadherin, keratin18.	Upregulated following CS exposure: fibronectin, vimentin. Downregulated following CS exposure: E-CADHERIN

S2 continued…

Reference	Exposure			Model		Findings	
	Type	Dose and time	Brand	Species	Cell type	Gene (mRNA)	Protein
Foronjy, R., et al., 2016	Cigarette smoke; Non-smoker and smoker	Mice 4h/d, 5d/wk for 6 months; Humans 35ml puffs and 6 puffs/cigarette for 4, 8 or 12 cigarettes	3R4F Kentucky	Mice and human	TLR9-/- mice and PTPLB-/- mice; normal human bronchial epithelial cells.	Upregulated following CS exposure: TLR9, CXCL5, G-CSF, MMP2, IL6, IL1BETA	Upregulated following CS exposure: TLR9
Fu, X., et al., 2009	Nicotine	1-500uM	N/A	NHP	Bronchial epithelial cells	Upregulated following nicotine exposure: nAChR.	
Gahring, L.C., et al., 2017	Cigarette	25-50 cigarettes for 225mins, 5d/wk for 4 months	3R4F Kentucky	Mice	α7G (CHRNA7 GFP tagged reporter) and α7E260A:G (homozygous receptor diminishes calcium current by 90% affecting developmental phenotypes and inflammatory responses)	Upregulated following CS exposure: SCGB1A1, ACTLB, CTSD, CTSS, VIM, CD74. Downregulated following CS exposure: CYP2F2	
Gao, H., et al., 2020	Smoke component BAP	48h 5ul/ml		Cell line	BEAS-2B	Upregulated following exposure to BaP: AhR, HSP90AA1, ARNT, CYP1A1, HIF-1ALPHA, TWIST1, TWIST2, SNAI2, BETA-CATENIN, N-CADHERIN, FIBRONECTIN, VIMENTIN, K-RAS. Downregulated following exposure to BaP: NRF2, E-CADHERIN.	

S2 continued…

Reference	Exposure			Model		Findings	
	Type	Dose and time	Brand	Species	Cell type	Gene (mRNA)	Protein
*Gebel, S., et al., 2006	Cigarette	300 or 600TPM/l 1h, twice/d, 5d/wk for 2, 7 or 13 wks	2R4F Kentucky	Rat	Lung	Upregulated following CS exposure: MT1a, MT2, FLAVIN, NR1D2, NQO1, ALDH3A1, CYP1A1, CXCL1, C3, DMBT1, MYBL1, FKBP38. Downregulated following CS exposure: ZMYND11, SPP1	Upregulated following CS exposure: Chitinase, NQO1, ALDH-3, EROD.
Geraghty, P., et al., 2014.	Cigarette	4hrs per day, 5 days a week for 1 day, 1 month, 2 months or a year		Mice and human	Human small epithelial cells	Upregulated following CS exposure: Src, c-Src, c-raf, MMP9, IL1BETA, IL6, TNF-ALPHA, MCP-1, CATHEPSINK, MMP12, MMP9, MMP12	Upregulated following CS exposure: P-ERK, P-JNK, P-P38
Ghosh, A., et al., 2021	Cigarette and pseudovirus	HBECs exposed to 14 puffs a day for either 1(acute) or 4 days(chronic). 3x10^4 U/ml pseudovirus. Varying concentrations of CSC (21.6-176uM nicotine conc) or JUUL tobacco-flavoured e-liquid (364-3,641uM nicotine conc) for 24hrs and infected with 2.5-5x10^5 U/ml pseudovirus.	1R6F	Human	Primary human bronchial epithelial cells; primary tracheobronchial cells and small airway epithelial cells	Upregulated following CS exposure: ACE2. Upregulated following SARS-CoV-2: ACE2.	Upregulated following CS exposure: ACE2.
Ginzkey, C., et al., 2012	Nicotine	0.001, 0.01, 0.1, 1.0, and 4.0mM		Human and cell line	Primary nasal mucosa and BEAS-2B cell line		

S2 continued…

Reference	Exposure			Model		Findings	
	Type	Dose and time	Brand	Species	Cell type	Gene (mRNA)	Protein
Golovatch, P., et al., 2009	Cigarette smoke	2 x 70ml puffs for whole body exposure 4h/d, 5d/wk for 1-12 wks	Kentucky 2RF reference cigarette	Guinea Pigs	Lung epithelial cells	Upregulated following CS exposure: JNK2, JNK1	Upregulated following CS exposure: P-ERK1/2, P-JNK1, P-JNK2, MAPK, Cathepsin K, MMP9. Downregulated following CS exposure: Elastin, collagen.
Gundavarapu, S., et al., 2012	Nicotine	6h/d, 5d/wk for 2wks. 1.5mg tpm.	N/A	Human	Normal bronchial epithelial cells	Upregulated following nicotine exposure: MUC5AC.	Upregulated following nicotine exposure: MUC5AC.
Guo, J., et al., 2005	Nicotine	0.5uM nicotine for various time periods	N/A	Rat	Lung epithelial cells		Upregulated following exposure to nicotine: Ras, cyclinD1, PKC, ROS, P13K, AKT
Haslbauer, J., et al., 2022	SARS-CoV-2	Infected patients		Human	Primary post mortem biopsy		Downregulated following SARS-CoV-2 exposure: AGTR1.
Hirschi-Budge, K. et al., 2022	Second-hand smoke exposure	The 10s primary puff is cleared and expelled so that the mice are only exposed to the side stream smoke three times/ 20 mins, 5d/wk	3R4F Cigarettes	Mice	C57BL/6 WT, RAGE knockout and transgenic	Upregulated following CS exposure: RAGE, Ras, NF-KB	Upregulated following CS exposure: RAGE, Ras, NF-kB, Cl-Caspase-3.
Ho, Y., et al., 2005	NNK tobacco component	Human cells 0-5uM; Mice 9.1mg	N/A	Human and mice	Normal human bronchial epithelial cells and small airway epithelial cells	Upregulated following CS exposure: CyclinD1, NFKB	Upregulated following CS exposure: CyclinD1, NFKB, P-ERK1/2, P38, CD1LUC, IKBALPHA, P-IKBALPHA, NFKB

S2 continued…

Reference	Exposure			Model		Findings	
	Type	Dose and time	Brand	Species	Cell type	Gene (mRNA)	Protein
Hou, W., et al., 2020	Cigarette smoke	CSE (10ug/ml, 20ug/ml and 50ug/ml) was introduced into the chip media for 8d and cells recovered in the absence of CSE for 7d		Cell line	BEAS2B	Upregulated following CS exposure: IL6, TNF-ALPHA, CLDN7, CLDN8. Downregulated following CS exposure: CLDN1, OCLD, BETA CATENIN, CDH1.	Upregulated following CS exposure: PHOSPHORYLATED STAT3, PHOSPHORYLATED ERK, C-MYC, CYCLIND1. Downregulated following CS exposure: E-CADHERIN, BETA CATENIN.
Hudlikar, R., et al., 2022.	NNK	24uMol/0.1ml		Mice	A/J female	Upregulated following CS exposure: NKRF, SOX14, CES3A, CYP3A25, UGT2B36, UGT2B5, CYP2J5, HRG, AKR1D1, BC024386, CCYP2C50, CYP2C54, JPH2. Downregulated following CS exposure: TRPC7, PDK2, ATXN1, SDR39U1, MPHOSPH8, FRAT1, TADA2A, TRAPPC4, CDK9, UKL1, NRARP.	
Hung, Y., et al., 2016	Cigarette (0.8mg nicotine)	CS 4 times/d for 1-3 weeks		Mice	Lung tissue		Upregulated following CS exosure: TGFBETA1, TNF-ALPHA, IL6, ACE, ACE2, P-P38, P-JNK, P-ERK1/2. Downregulated following CS exposure: MMP2, MMP9.
Irie, H., et al., 2022	Cigarette	60min/d, 5d/wk for 3wk	Malboro (12mg tar/ 1mg nicotine)	Mice	C57BL6J; GFP and surfactant protein C expressing mice	Upregulated following CS exposure: GPX1, GCLM, GCLC.	Upregulated following CS exposure: CPT1A.

S2 continued…

Reference	Exposure			Model		Findings	
	Type	Dose and time	Brand	Species	Cell type	Gene (mRNA)	Protein
Irie, H., et al., 2022	CS with nicotine	Mice 60min/d, 5d/wk	Malboro (12mg tar/1.0mg nicotine	Mice	C57BL6J mice and mice expressing GFP in surfactant protein C	Upregulated following CS exposure: CPT1A.	
Izzotti, A., et al., 2010	Environmental cigarette smoke	28 days		Rats	Lungs	Downregulated following CS exposure: NF-KBETA, P53, TGF-BETA, ELK-1, FOS	
Jiang, J.-X., 2017	Cigarette smoke	10 cigarettes, 1.5hr whole body exposure	Kentucky 3R4F reference cigarette	Cell line and mice	16HBE and C57BL/6 mice	Upregulated following CS exposure: IL6, IL8, RAC1	Upregulated following CS exposure: RAC1, IL6, IL8, CD68, CD11B, ERK1/2, STAT3
Lallai, V., Manca, L. and Fowler, C., 2021	E-cigarette and nicotine	1 puff every 5 mins for an hour for 5 days with 7.5mg/mL nicotine or without		Mice	C57BL/6 J WT lungs	Upregulated following e-cig with nicotine: ACE2. Downregulated following e-cig with nicotine: alpha5 nAChR.	Upregulated following e-cig with nicotine: ACE2.
Lam, D., et al., 2016	Nicotine	Never smoker, active smoker or ex-smoker. 100uM nicotine.	N/A	Human and cell line	Human bronchial epithelial cells and HBEC-KT 2-5 cell lines	Upregulated following nicotine exposure: CHRNA5, CHRNA7, CHRNA6. Downregulated following CS exposure: CHRNA6.	
Latha, M., et al., 1991	Cigarette smoke	Exposed twice/d for 90d for 15mins(day 1), 30mins(day 2), 45mins(day 3) and 1hr(day 4+)		Rats	Lungs	Upregulated following CS exposure: Glycosaminoglycans, CTSD, PARG, GUSB	
Lee, A., et al., 2020	Smoking	Current smoker or former smoker	N/A	Human		Upregulated following CS exposure: ACE2	

S2 continued…

Reference	Exposure			Model		Findings	
	Type	Dose and time	Brand	Species	Cell type	Gene (mRNA)	Protein
Lee, H.S. and J. Kim, 2013	Cigarette condensate	2 cigarettes smoked consecutively over 10 minutes bubbled through 10mL media and CSE was used at 0.3-300ug/ml	2R4F	Human	Primary nasal epithelial cells		
Lee, I., et al., 2020	Cigarette / SARS-CoV-2	Former, current or never smoker	-	Human and mice	Human nasal tissue, lung tissue	Upregulated following SARS-CoV-2 exposure. No significant difference in ACE2 or TMPRSS2 following CS exposure.	
Lemjabbar, H., et al., 2003	Smoke exposures	1mg smoke particulate per ml		Mice and Human	C57 mice; primary human airway epithelial cells		Upregulated following CS exposure: P-EGFR. Downregulated following CS exposure: ADAM17.
Leung, J., et al., 2020	Cigarette	Current, former or never smoker samples	N/A	Human	Primary bronchial epithelial cells	Upregulated following exposure to CS: CHRNA7, ACE2	
Li, D., et al., 2022	SARS-CoV-2 pseudovirus	Transocular inoculation of mice		Mice	ACE2 dependence, humanised ACE2 and ACE2 KO mice	Upregulated following SARS-CoV-2 exposure: ACE2.	
Li, E., et al., 2014	Cigarette	CSE(1-100% exposure) for varying lengths of time (1 hr - 8 wks).		Human	Primary bronchial epithelial cells adjacent to a cancer tumour		Upregulated following CS exposure: VIMENTIN, GRP78, IRE1ALPHA, P13K/AKT, MTOR, IKKALPHA, NF-KB, CYCLIND1. Downregulated following CS exposure: E-CADHERIN.

S2 continued…

Reference	Exposure			Model		Findings	
	Type	Dose and time	Brand	Species	Cell type	Gene (mRNA)	Protein
Li, F., et al., 2012	Smoking	7 cigarettes (day1), 9 cigarettes (day 2) and 11 cigarettes (day 3 and 4).	3R4F Kentucky	Mice	C57BL/6	Upregulated following CS exposure: Shp2, IL8.	Upregulated following CS exposure: Shp2, IL8, MMP2, KC
Li, F., et al., 2021	SARS-CoV-2 pseudovirus	MOI of 5 for 24h		Human cell line; Mice	16HBE; BALB/c mice		Upregulated following SARS-CoV-2 exposure: LC3-II, BAX, IL6, IL8, TNF-ALPHA. Downregulated following SARS-CoV-2 exposure: p62, BCL-2, mTOR, AKT.
Li, G., et al., 2020	Smoking and SARS	12, 24 and 48h post SARS infection; before and after smoking 3 cigarettes in 24h		Human	Lung tissue, BAL, bronchial epithelial cells, small epithelial cells and SARS infected cells	Upregulated following CS exposure: ACE2. Upregulated following SARS-CoV-2 exposure: ACE2, IL6, IL10, IL1.	
Li, H., et al., 2021	Cigarette	Smoker vs non-smoker - CSE exposure for 48hrs	N/A	Human	Bronchial epithelial cells	Upregulated following CS exposure: TROP2, P38, P65, IL6, IL8, VIMENTIN. Downregulated following CS exposure: E-CADHEIRIN	Upregulated following CS exposure: TROP2, P38
Li, Q., et al., 2010	Cigarette smoke	10 cigarettes, 10 puffs/cigarette, 1 puff/min into 20mls buffer	Camel cigarettes	Cell line	BEAS2B	Upregulated following CS exposure: ROS, MUC5AC	Upregulated following CS exposure: MUC5AC, SRC, EGFR, P-ERK, JNK
Li, Q., et al., 2011	Cigarette smoke and Nicotine	100ug/ml CSE or 20uM nicotine for 24h	Camel brand cigarette	Cell line	HBE16	Upregulated following CS exposure: MyD88. Downregulated following nicotine exposure: TNF-ALPHA.	Upregulated following CS exposure: MyD88, 1-Kbalpha, P65. Downregulated following nicotine exposure: TNF-ALPHA, P65.

S2 continued…

Reference	Exposure			Model		Findings	
	Type	Dose and time	Brand	Species	Cell type	Gene (mRNA)	Protein
Li, Q., et al., 2012	Cigarette and nicotine	CS for 12hr; Nicotine conc 5, 10, 20uM	Camel brand	Cell line	HBE16	Upregulated following nicotine exposure: CHRNA1, CHRNA5, CHRNA7, CHRNB2, TNF-ALPHA, IL8, IL6.	Upregulated following nicotine exposure: CHRNA1, CHRNA5, CHRNA7, TNF-ALPHA, IL6, IL8.
Li, W., et al., 2021	Smoking	Smoker vs Non-smoker	N/A	Human	Nasal mucosa, bronchial mucosa and pulmonary alveoli	Downregulated following CS exposure: ACE2	Downregulated following CS exposure: ACE2
Lin, C., et al., 2021	Cigarette	Current, former or never smoker samples	N/A	Human	AT2 cells from donors	Upregulated following CS exposure: CD147, HERPUD1, GRP78, CD209L, ACE2, TMPRSS2,	Upregulated following CS exposure: CD147, HERPUD1, GRP78, CD209L, ACE2, TMPRSS2
Liu, A., et al., 2021	Cigarette smoke	Non-smoker, current smoker and ex-smoker; Mice exposed to 5 cigarettes, 4 times per day, 6 days/week for 1, 4 or 8 months.	Unknown but 14mg tar and 1mg of nicotine per cigarette	Human and mice	Small epithelial cells and type II alveolar cells	Upregulated following CS exposure: HIF-1alpha, iNOS, 4HNE, ACE2	Upregulated following CS exposure: ACE2. Downregulated following CS exposure: ACE2.
Lupacchini, L., et al., 2020	Nicotine	1x10^7M nicotine for 1h or 48h	N/A	Human	Bronchial epithelial cells		Upregulated following nicotine exposure: EGFR, PHOSPHO EGFR, PHOSPHO38, VEGF, VIMENTIN, FIBRONECTIN, VEGF, KI67, CA2+, ACE2, ALPHA7 NACHR. Downregulated following nicotine exposure: ATP, P53, PHOSPHO P53, E-CADHERIN.

S2 continued…

Reference	Exposure			Model		Findings	
	Type	Dose and time	Brand	Species	Cell type	Gene (mRNA)	Protein
Lv, J., et al., 2021	SARS-CoV-2	Increased viral load and measured infection at 2hr or 72hr and control (no infection)		Cell line, murine and NHP	BEAS-2B and murine alveolar epithelial type II cells	Upregulated following SARS-CoV-2 exposure: AhR, ACE2.	
Ma, L., et al., 2020	Cigarette	24h at 20%CSE		Mice	Bronchial epithelial cells	Upregulated following CS exposure: IL17R, VIMENTIN. Downregulation following CS exposure: E-CADHERIN	Upregulated following CS exposure: NF-KB
Maehira, F., et al., 1999	Cigarette	Via ash tray 10cm below rat cage for 1hr twice/d for 8, 12 or 20 wks		Rat	lungs	Upregulated following CS exposure: KRAS, C-PKC. Downregulated following CS exposure: PKC, C-PKC.	Upregulated following CS exposure: 8OHDG. Downregulated following CS exposure: KINASEA
Mao, Y. and Feng, H., 2022	CSC	5, 10 or 20% cigarette smoke concentrations	15 lit cigarettes (Sichuan China Tobacco Industry Co., Ltd.)	Human	16-HBE cell line		Upregulated following CS exposure: slug, alpha DMA, collagen IV, FN1, TGF-beta1, p-SMAD3. Downregulated following CS exposure: e-cadherin, CC16.

S2 continued…

Reference	Exposure			Model		Findings	
	Type	Dose and time	Brand	Species	Cell type	Gene (mRNA)	Protein
Marshall, K., et al., 2020	E-cig and nicotine	e-cig aerosol (18mg/mL nicotine) exposure for 8 months		Mice	Lungs	Upregulated following CS exposure: BCL-XL. Upregulated following e-cig with nicotine exposure: BCL-XL. Downregulated following CS exposure: E-CADHERIN, CRM1. Downregulated following e-cig with nicotine exposure: E-CADHERIN, CRM1.	Upregulated following CS exposure: CYP1A1, CYP2A5, AhR, NRF2, SOD1, BCL-XL, P21. Upregulated following e-cig with nicotine exposure: CYP1A1, CYP2A5, AhR, NRF2, SOD1, BCL-XL. Downregulated following CS exposure: E-CADHERIN, CRM1. Downregulated following e-cig with nicotine exposure: E-CADHERIN, CRM1.
Martinez-Garcia, E., et al., 2008	Nicotine	1 to 500uM nicotine daily for 7 days		Human	Normal bronchial epithelial cells	Upregulated following nicotine exposure: N-CAM, Nef-M, Pax-3, alpha7 nAChR. Downregulated following nicotine exposure: e-cadherin, ZO-1, beta2 nAChR.	Upregulated following nicotine exposure: EGFR.
Masso-Silva, J., et al., 2021	E-cig and nicotine	Vape pen (24mg/ml with nicotine) / box (with or without 6mg/ml nicotine) 60min/d for 3-6 months; JUUL exposure (with 59mg/ml nicotine)20 mins 3 times/d for 1 month		Mice	Lungs	Upregulated following e-cig with nicotine exposure: KRT83. Downregulated following e-cig with nicotine exposure: CD177, FACMR, TLR9, FCGRL, CCR2, IL6, IL1, IL2, TNF, IFNGAMMA, TGFBETA, P13K, JAK/STAT, ERK1, ERK2, IKB, NFKB, CD40, ACE2, AGT, SLC7.	

S2 continued…

Reference	Exposure			Model		Findings	
	Type	Dose and time	Brand	Species	Cell type	Gene (mRNA)	Protein
McAlinden, K., et al., 2021	E-cig with or without nicotine, PG:VG or CSE	E-cig condensate (0.05%, 0.1%, 0.5% or 1% conc) with or without nicotine (18mg/mL or 60mg/mL); CSE (0.5% or 1% conc).	Watermelon and menthol flavoured e-liquid(Juicius Maximus, VapeTrail); 1R6F Kentucky Tobacco Research, USA	Human	BEAS-2B cell line and primary small airway epithelial cells	Upregulated following CS exposure: ACE2. Upregulated following e-cig with nicotine exposure: ACE2.	Upregulated following CS exposure: ACE2. Upregulated following e-cig with nicotine exposure: ACE2.
Mebratu, Y., et al., 2011	Cigarette	Mice exposed to 250mg/m^3 CS for 6h/d, 5d/wk for 3 wks		Human and Mice	Primary airway epithelial cells, ; Mice C57BL/6, Mouse airway epithelial cells	Upregulated following CS exposure: MUC5AC. Downregulated following CS exposure: BIK	Upregulated following CS exposure: STAT1, ERK1/2
Mishra, R., et al., 2016	Cigarette	Cells CSE exposed for 10 minutes; Mice 6h/day, 5d/wk	2R4F	Human and mice	Primary alveolar epithelial cells; NuLi-1 cells (nontransformed human airway epithelial cell line and normal human bronchial epithelial) Mice C57B16/J.		Upregulated following CS exposure: HER2, PHER2, HER3, pHER3, pEGFR, EGFR, IL6.
Monzon, M., et al., 2011.	Cigarette	Smoker or non-smoker	N/A	Human	Normal bronchial epithelial and tracheobronchial		Upregulated following CS exposure: MUC5AC, MUC5B, MCP-1, CCR2B

S2 continued…

Reference	Exposure			Model		Findings	
	Type	Dose and time	Brand	Species	Cell type	Gene (mRNA)	Protein
Nakayama, T., et al., 2021	Cigarette and SARS-CoV-2	CSE 1hr/d for 3d before SARS-CoV-2 inoculation; Viral infection exposure involved 100ul of viral suspension 5-10x10^5PFU/mL for 2hrs	3R4F University of Kentucky (9.4mg tar/ 0.726mg nicotine)	Human	Nasal epithelial cells and COVID-19 infected patient samples		Upregulated following CS exposure: IFNB1.
Nath, S., et al., 2019	Cigarette	4h/d, 5d/wk	3R4F Cigarettes	Mice	C57BL/6J CTSS knockout mice	Upregulated following CS exposure: CTSE, CTSS, CTSG.	Upregulation following CS exposure: CTSS
O'Donnell, R. et al., 2004	Smoker vs non-smoker tissues			Human	Bronchoscopy tissues		Upregulated following CS exposure: EGFR, ERBB3, MUC5AC.
Ogawa, F., et al., 2019	Cigarette smoke	CSE from one cigarette bubbled through 12.5ml media	Malboro Red cigarette	Human	Non-smoker primary basal cells	Upregulated following CS exposure: KRAS activation, RAS	Upregulated following CS exposure: KRAS, RAS
Osborne, J. et al., 2014	Nicotine	Increasing conc for 24-48h	N/A	Human cell line	HBEC3KT-53; HBEC3KT, HBEC4KT, HBEC30KT	Upregulated following nicotine exposure: NEUROD1, CHRNA3, CHRNA5, CHRNA7, CHRNB2, CHRNB4.	Upregulated following CS exposure: NEUROD1. Downregulated following CS exposure: p53.
Peng, Y., et al., 2022	Smoker	Smoker vs non-smoker samples		Human	Primary small airway epithelial cells	Upregulated following CS exposure: ACE2, TMPRSS2. Downregulated following CS exposure: BSG, ITGAV.	

S2 continued…

Reference	Exposure			Model		Findings	
	Type	Dose and time	Brand	Species	Cell type	Gene (mRNA)	Protein
Phandthong, R., et al., 2022	E-cig	Vitrocell: 1 puff of PBS nicotine (0.3 or 0.003mg/ml) during a 1.5 mins aerosol generation followed by 3 min aerosol deposition. CULTEX: 55ml air or e-cig aerosol for a 4s puff with a 30s interval	JUUL aerosols	Cell line	BEAS-2B and HEK293T		Upregulated following nicotine exposure: ACE2, TMPRSS2. Upregulated following e-cig with nicotine exposure: ACE2, TMPRSS2. Upregulated with e-cig: ACE2. Downregulated with e-cig: TMPRSS2.
Podowski, M., et al., 2012	Cigarette	Mice exposed 2h/day, 5 d/wk, 6/7 wks and MLE12 cells treated with CSE for 72hrs		Mice	Lung tissue	Upregulated following CS exposure: MMP9, MMP12. Downregulation following CS exposure: MAPK, AKT, P38, JNK	Upregulation following CS exposure: TGF-BETA, SMAD2, CTGF
*Polk, W., 2012	Cigarette	CSC from 20 cigarettes with final conc 40mg/mL	3R4F Kentucky Reference	Cell line	BEAS2B	Upregulated following CS exposure: MMP3, MAP1B. Downregulated following CS exposure: KRT7, KRT9, SNAI3, MMP2	Upregulated following CS exposure: BETA-CATENIN, E-CADHERIN
Polosukhin, V., et al., 2011	Cigarette	Current smoker or never smoker	N/A	Human	Lung tissue and bronchial epithelial cells	Upregulated following exposure to CS: MUC5AC, CA IX	Upregulated following exposure to CS: MUC5AC, ERK, HIF-1ALPHA, P-ERK1/2
Profita, M., et al., 2011	Cigarette	18h with 10% CSE	Commercial cigarettes (Marlboro; Philip Morris USA, Richmond, VA)	Cell line	16-HBE	Upregulated following exposure to CS: ChAT, ACh, LTB4, M2, M3	Upregulated following exposure to CS: ChAT, ACh, LTB4, M2, M3

S2 continued…

Reference	Exposure			Model		Findings	
	Type	Dose and time	Brand	Species	Cell type	Gene (mRNA)	Protein
Qiao, Y., et al., 2021	Cigarette smoke	Smoker vs non-smoker	N/A	Human and mice	Lung tissues		Upregulated following CS exposure: ACE2
Rangasamy, T., et al., 2009	Cigarette	Mice exposed to CS 7 hrs/d, for 1 day(Group II), 8 days (Group III), 1.5 months (Group IV) or 6 months (Group V)	2R4F (2.45mg nicotine per cigarette)	Mice	A/J mice	Upregulated following CS exposure: CYP1A1, CYPB1B1, ADAM8, CTSB, CTSK, MAP2K6.	
Rayner, R., et al., 2022	Smoking and e-cigarette	100ul exposure of whole-smoke conditioned media/e-liquid aerosol conditioned media at various nicotine concentrations for 1h/day for 10 days	3R4F cigarette and commercially available e-liquid 2ith 1.8% nicotine	Human	Primary human bronchial epithelial cells	Upregulated following CS exposure: CYP1A1, CYP1B1, ALDH3A1, AKR1C1, AKR1C2, GXP2, NQO1, FTL, ADH7, CEACAM5, MUCL1, MUC5AC, KRT13, LUCAT1, OSGIN1, MAGEA6, ABCB6, SLC9A5, IL36A. Upregulated following e-cig with nicotine exposure: CYP1B1, MAGEA6, ABCB6, SLC9A5. Downregulated following CS exposure: EN1, CXCL10, CXCL5, CSF3, CCL20.	
Russo, P., et al., 2020	Nicotine	1 x 10^-7M (concentration present in alveolar lining fluids after one cigarette is between 6x10^-6 and 6x10^-5) for 1hr or every 58 hrs for 16 passages.		Human and cell line	Primary bronchial epithelial cells and HBE16	Upregulated following nicotine exposure: ACE2, PHOSPHO-S6 RIBOSOMAL PROTEIN, AKT1, PHOSPHO-AKT, PHOSPHO-P44, P43, MAPK	

S2 continued…

Reference	Exposure			Model		Findings	
	Type	Dose and time	Brand	Species	Cell type	Gene (mRNA)	Protein
Sarkar, P. and Hayes, B., 2007	Tobacco - acrolein	Incubated for 72h with different acrolein concentrations (0-45uM)	N/A	Rat	Lung epithelial cells	Upregulated following CS exposure: COX-2, NF-KB, CA+2, ERK, MEKK	Upregulated following CS exposure: RAF-1
Shaykhiev, R., et al., 2013	Cigarette	Smoker or non-smoker; ALI cells exposed to 1-2% CSE for 24hrs every other day for 2 wks.	Malboro Red cigarette	Human	Large airway epithelial cells	Upregulated following exposure to CS: ERBB4, EGF, NRF-2, KRT6, KRT14, IVL, SFN, CD44, SNA12, VIMENTIN. Downregulated following exposure to: EGFR, ERBB2, TGF-ALPHA, KRT5, FOXJ1, DNAI1, MUC5B, SCGB1A1, E-CADHERIN, TJP1, CLDN, OCLN, PARD3, PARD6, PTEN	Upregulated following CS exposure: EGF. Downregulated following CS exposure: EGFR.
Shen, H., et al., 2022	CS with nicotine	16HBE CSE exposure length unknown; Mice exposed to 10 cigarettes/d for 20d	3R4F Kentucky University	Human and mice	16HBE cell line; C57BL/6 mice	Upregulated following CS exposure: mAChRM3, ChAT, VIM, alphaSMA, Akt, p-Akt. Downregulated following CS exposure: E cadherin.	Upregulated following CS exposure: ChAT, Ach, VIM, alphaSMA. Downregulated following CS exposure: E cadherin.
Sompel, K., et al., 2022	CSC	HBEC cells after 24hr 5ug/ml of CSC, 2/wk for 1-24 weeks; Mice whole body exposure for 6h/d, 5d/wk for 6wks		Human and mice	HBEC3KT and HBEC2KT cells; FZD9 -/- and FVBN mice	Upregulated following CS exposure: VIM. Downregulated following CS exposure: FZD9, ECAD, PPARgamma, PPRE	Upregulated following CS exposure: VIM. Downregulated following CS exposure: ECAD

S2 continued…

Reference	Exposure			Model		Findings	
	Type	Dose and time	Brand	Species	Cell type	Gene (mRNA)	Protein
Staudt, M., et al., 2018	E-cig and nicotine	10 puffs, wait 30m before another 10 puffs		Humans	Small airway epithelium	Upregulated following e-cig with nicotine exposure: LINC01186, AJUBA, LATS2, AMOTL2, SERPINB2, CST6, RND3. Upregulated following e-cig: LTB4R2, ISG20, GRHL3, TXK, LGALS7B, SLC5A8, BATF3. Downregulated following e-cig with nicotine exposure: BEST3, MYL5, MTIX, SGK1, MT2A, NRIDI, NEURL3. Downregulated following e-cig exposure: DNASEIL2, TMEM220-ASI, TMEM171, PLXDC1, CALML5.	
Tan, L., et al., 2018	Cigarette	Smokers smoker 10-20 cigarettes/d for 3 years; Mice CS exposure 2hrs/d for 3 wks.	3R4F (University of Kentucky)	Human and mice	ATII cells from organ donors; C57BL/6 mice	Upregulated following CS exposure: ADAMTSL4	Upregulated following CS exposure: MMP9, CD147, ADAMTSL4. Downregulated following CS exposure: P18 NFE2, P45 NFE2.
Tang, X., et al., 2021	Cigarette	Human cells: 2.5% CSE for 24h; Rats: 15 cigarettes, 30 min each twice/d, 6d/wk for 16wk	Daqianmen cigarettes (10mg tar, 1mg nicotine and 12mg CO)	Human and Rats	BEAS-2B cell line; Sprague-Dawley rats		Upregulated following CS exposure: IL6, TNFALPHA, MDA, IRON, TFR1. Downregulated following CS exposure: GSH, SLC7A11, GPX4, FTH1.

S2 continued…

Reference	Exposure			Model		Findings	
	Type	Dose and time	Brand	Species	Cell type	Gene (mRNA)	Protein
Tomchaney, M., et al., 2020	Smoking and SARS-CoV-2	Human lung specimen: smoker or non-smoker; Mice: 2h/d, once every 6d for 1-6 months; Primary HBEC: CSE and 5min cigarette smoke and infected with SARS-CoV-2 at an MOI of 0.05 PFU/cell.	3R4F Cigarettes	Human	Lung specimens from smokers without COPD and non-smokers; C57BL/6 mice; Primary human bronchial epithelial cells		Upregulated following CS exposure: GammaH2AX. Downregulated following CS exposure: ACE2, MUC5AC.
Tsutsumi, A., et al., 2020	Cigarette	CS via nasal inhalation 60min/d, 5d/wk for 12 wks.	Malboro (12mg tar/ 1mg nicotine)	Mice	C57BL6 mice	Upregulated following CS exposure: DBP, CFB, NR1D1, VNN1, LY6I, STEAP4, PIGR, NR1D2, TNFRS19, ST6GALNAC3. Downregulated following CS exposure: RETNLA, NPAS2, ARNTL, HMGCS2, CCNJ1, CLCA1, AVPR1A, DEFB5, RASL11A, KLH13.	
*Van der Toorn, M., et al., 2017	Cigarette	Continuous exposure for 12 wks (Low (7.5ug/mL TPM; 2.5uM nicotine), medium (37.5ug/mL TPM; 6uM nicotine) or high (150ug/mL; 24uM nicotine)). Note nicotine concentration much lower in THS compared to 3R4F.	3R4F (University of Kentucky) and tobacco heating system 2.2	Cell line and human	BEAS-2B and primary normal bronchial epithelial cells	Upregulated following exposure to CS: IL1A, IL8, CXCL8, CSF-2, CSF-3, VEGFA, MMP1, MMP9, IL6 TIMP1. Downregulated following exposure to: CCL5, CXCL10, TIMP2	Upregulated following CS exposure: vimentim, mBCL, PH2AX. Downregulated following CS exposure: e-cadherin, DHE.

S2 continued…

Reference	Exposure			Model		Findings	
	Type	Dose and time	Brand	Species	Cell type	Gene (mRNA)	Protein
*Veljkovic, E., et al., 2011	Cigarette	20ug/ml of CSC (0.002 cigarettes/ml)		Cell line	BEAS-2B	Upregulated following CS exposure: PAK3, ADAM19, AGTR1, TGFB1, TGFB2, ADAMTS3, ASAMTS12, MMP1. Downregulated following CS exposure: E-CADHERIN, EGF, G-TYPE RECEPTOR, EPPK1, MAP7, BIK, MMP14, MUC1, EVA1, NRP3, FOS, TNSF10, MMP7, MUC20, TMPRSS3	
Vivarelli, F., et al., 2021	Cigarette	2s puff with 35ml volume and 30s intervals for 20 mins never for more than 3h/d. It was 4d/wk for 4 wks		Rats	Sprague Dawley rats	Upregulated following CS exposure: IL-13, IL-10, IL-12, TNF-alpha, INF-gamma, CSF2, CCL3, COX, CYP1A1, CYP2A1/2, CYP2B1/2, CYP2E1, JNK, ERK1/2, p38 MAPK.	Upregulated following CS exposure: 8-oxo-dG
Voinsky, I. and D. Gurwitz, 2020	Smoking	Smokers vs non-smokers	N/A	Human	Bronchial epithelial cells	Upregulated following CS exposure: TMPRSS4, NQO1, ALDH3A1, GPX2, AKR1C2, PIR, MUC5AC, ME1, CLDN10, CX3CL1, TXN, PPP1R16B, FAM114A1, SRPX2, S100A10, TRIM16, UGT1A1	

S2 continued…

Reference	Exposure			Model		Findings	
	Type	Dose and time	Brand	Species	Cell type	Gene (mRNA)	Protein
Wang, G., et al., 2021	Cigarette, nicotine, BaP, NNK and pseudovirus	CSE(5-20%) for 0-72hrs; Nicotine 2.5-10umol/L for 0-72hrs; NNK at 5umol/L for 48hrs; BaP at 5umol/L for 48hrs; Mice exposed to CS via 750ug TPM/L for 20d, BaP at 100mg/kg twice a wk for 5 ks and NNK treatment at 50mg/kg twice a wk for 5 wks. Exposure with Pseudovirions involves incubation with 100uL of media for 48hrs.		Human and mice	16HBE cell line, BEAS2B cell line and lung samples (tissue adjacent to lung cancer); Mice A/J	Upregulated following CS exposure: ACE2, IL1ALPHA, IL-1BETA, IL-2, IL-6, TNFALPHA, SKP2. Downregulated following CS exposure: ACE2.	Upregulated following CS exposure: SKP2. Downregulated following CS exposure: ACE2.
Wang, Q., et al., 2014	CSE	1-5% CSE for up to 5d	13mg tar; 1.2mg nicotine per cigarette	Human	BEAS-2B cell line	Upregulated following CS exposure: uPAR, AKT.	Upregulated following CS exposure: N-cadherin, Vimentin, Alpha-SMA, uPAR, p-AKT. Downregulated following CS exposure: E-cadherin.
Wang, Q., et al., 2020	E-cig with or without nicotine	3.3s/puff, twice per minute with 70mL puff volume. Whole body exposure for 2hrs/d, 5d/wk for 30d with a 2.0L/min flowrate in the exposure chamber	Joytech VTC mini (SciReq, Montreal Canada)	Mice	C57BL/6J mice; nAChR α7 KO (α7 nicotinic acetylcholine receptor knockout mice); nAChR α7 CreCC10 mice (clara/club-cell-specific nAChR α7 deletion)	Upregulated following e-cig with nicotine: MMP8, TMP3. Downregulated following e-cig with nicotine: NECTIN1, SKIL, LDLR, COL1A2, COL4A1. Downregulated following e-cig: MMP8, MMP9, COL1A2, COL4A1.	Upregulated following e-cig with nicotine: p50, p105, ACE2, MMP9, MMP2, MMP12, PA1-1. Upregulated following e-cig exposure: MMP2. Downregulated following e-cig with nicotine: COL1A1. Downregulated following e-cig alone: ACE2, MMP8, MMP9, fibronectin.

S2 continued…

Reference	Exposure			Model		Findings	
	Type	Dose and time	Brand	Species	Cell type	Gene (mRNA)	Protein
Wang, Y., et al., 2001	Nicotine	1uM nicotine for 3 days	N/A	Human	Bronchial epithelial cells and airway epithelial cells	Upregulated following nicotine exposure: CHRNA7	Upregulated following nicotine exposure: CHRNA7
Wark, P., et al., 2021	Cigarette	Pack years smoked	-	Human	Primary bronchial epithelial cells	No significant difference in any genes	
West, K., et al., 2003	Nicotine or NNK	10uM nicotine for 60mins; 1uM NNK added for 45mins	N/A	Human	Normal bronchial epithelial cells and small airway epithelial cells	Upregulated following CS exposure: CHRNA7. Upregulated following nicotine exposure: CHRNA3, CHRNA4, GSK-3, P70S6K, 4EBP1, FKHR.	Upregulated following CS exposure: AKT. Upregulated following nicotine exposure: P-AKT, P13K.
Wick, K. et al., 2022	E-cig	10puffs/min for 20-60min/d for 3d.	JUUL aerosol (Mint flavoured JUUL pod containing 0.7ml 5% nicotine salt e-liquid)	Human	Primary AT2 cells	Upregulated following e-cig with nicotine exposure: SLC7A11, SLC5A3, SLC38A2, CYP1A1, CYP1B1, AKR1C2, PGM2L1. Downregulated following e-cig with nicotine exposure: CCL2, CXCL2, EGR1, CFTR, JUN, ZNF385B, NOD1, SRSF5, FOS.	

S2 continued…

Reference	Exposure			Model		Findings	
	Type	Dose and time	Brand	Species	Cell type	Gene (mRNA)	Protein
Wohnhaas, C., et al., 2021	Cigarette	Before differentiation CSE 4 cigarettes, 3/wk for 28d; after differentiation CSE was on four consecutive days	3R4F	Human	Small airway epithelial cells	Upregulation following CS exposure: CYP1A1, CYP1B1, ALDH1A3, MGST1, GPX2, AKR1C2, AKR1C1, AKR1C3, AKR1B10, TKT, ALDH3A1, ADH7, TALDO1, GCLC, TXN, GSTP1, PRDX1, NQO1, FTL, FTH1, MUC1, CXCL17, CXCL8, RARRES2, S100A8, S100A9, RNASE1, LCN2, BP1FA2, GPX2, GPX1, SAA1, SAA2, EGFR, TMPRSS4. Downregulation following CS exposure: CYP2F1	Upregulated following CS exposure: MUC5AC
Yang, J., et al., 2017	Smoking	Smokers vs non-smokers	N/A	Human	Proximal airway vs distal airway	Upregulated following CS exposure: UPK1B, GPX2, CYP26A1, CD44, ADH7, CYP4F3, IL1R2. Downregulated following CS exposure: LTF, SCGB3A2, SFPTB, MGP, WIF, TACR1, FOLR1, SOX9, TLR4, FOXA2, ARX, NPR3.	
Yang, J., et al., 2021	SARS-CoV-2 pseudovirus and cigarette	6h incubation at 37 degrees; CS	N/A	Human and mice	BEAS-2B cell line and MLE-12	Upregulated following CS exposure: ACE2. Downregulated following CS exposure: MIZ1	Upregulated following CS exposure: ACE2.
Yu, H., et al., 2011	Cigarette smoke	50mls one puff/min	N/A	Cell line	16HBE	Upregulated following CS exposure: NRG1BETA, ERBB3, MUC5AC	Upregulated following CS exposure: NRG1BETA, ERBB3, MUC5AC, P-ERK, JNK, TACE

S2 continued…

Reference	Exposure			Model		Findings	
	Type	Dose and time	Brand	Species	Cell type	Gene (mRNA)	Protein
Yu, X., et al., 1992	Cigarette	No exposure vs acute cigarette smoke exposure for 5 mins or chronic cigarette smoke exposure 1hr/d for 30d.		Rats	Lungs	Upregulated following CS exposure: ACE.	
Zhang, L., et al., 2013	Cigarette smoke	8 x 35ml puffs for 2s/1min/cigarette, cell culture medium. Cell culture medium exposed to smoke for 6 continuous hrs	Kentucky 3R4F reference cigarette	Human	Primary human bronchial epithelial cells	Upregulated following CS exposure: RAC1, CDC42, P120CTN.	
Zhang, L., et al., 2013	Cigarette condensate	4h		Human	Primary Human bronchial epithelial cells		Upregulated following CS exposure: TyrP EGFR, pSrc, p-JNK. Downregulated following CS exposure: MUC1-CT, P120CTN, BETA-CTN, E-CADHERIN, p-AKT.
Zhang, L., et al., 2014	Cigarette smoke	cigarette condensate		Human	Primary human bronchial epithelial cells		Upregulated following CS exposure: MUC1-N. Downregulated following CS exposure: Cadherin, p120ctn.
Zhang, L., et al., 2016	Cigarette condensate	6 hours of cigarette burning		Human	Bronchial epithelial cells	Upregulated following CS exposure: Fos, Fra1, FosB	Upregulated following CS exposure: MUC1-CT, P120CTN, ERK-P

S2 continued…

Reference	Exposure			Model		Findings	
	Type	Dose and time	Brand	Species	Cell type	Gene (mRNA)	Protein
Zhang, Y., et al., 2021.	Cigarette	5 commercial cigarettes for two 30min sessions/d for 4 wks		Mice	C57 mice MLE-12 (AT2 cell line and AT2 cells	Upregulated following CS exposure: p21, p16, PARAP1. Downregulated following CS exposure: SIRT1, COXIV, LC3, LAMP1.	Upregulated following CS exposure: P16, P21, LC3.
Zhao, Y., et al., 2013	CSE	0-100ug/ml for 24h, 48h or 20wk	1R4F (9mg Tar; 0.8mg Nicotine per cigarette)	Human	HBE cell line	Upregulated following CS exposure: IL6.	Upregulated following CS exposure: N-cadherin, vimentin, p-p65, IL6, NF-KB. Downregulated following CS exposure: E-cadherin.
Zia, S., et al., 1997	Nicotine and cigarette smoke	0, 10uM, or 1mM of nicotine for 24hrs; Non-smoker and heavy smoker	N/A	Human and mice	Bronchial epithelial cells and mice lungs	Upregulated following CS exposure: CHRNA3. Upregulated following nicotine exposure: CHRNA7, CHRNA3, CHRNA5, CHRNA4.	Upregulated following CS exposure: Ca2+. Upregulated following nicotine exposure: CHRNA5.

* Studies declare funding from tobacco and/or e-cigarette manufacturers.

Abbreviations: CS = Cigarette smoke; E-cig = Electronic cigarette; h(rs) = hour(s); d = day; wk= week; CSC = Cigarette smoke condensate; CSE = Cigarette smoke extract.

Supplementary information 3: Quality scoring tool. The six domains included studies were scored against. Higher scores were assigned to studies investigating core viral-entry genes (ACE2 and TMPRSS2) as this is a key factor for addressing the aims of this study.

	Cell model		Route of exposure		Dosage	Gene and protein expression		Cytotoxicity		SARS-CoV-2 challenge
	In vitro	In vivo	In vitro	In vivo		Assays	Genes	In vitro	In vivo	
**HIGH (3)**	Primary human epithelial cells at ALI for 14+ days	NHP Animal Model OR tissues	Aerosolised exposure to cells	Intranasal exposure	Chosen dosage related to human exposure with appropriate controls or dose dependent curve with relevant controls	Studies followed up gene expression studies with multiple protein expression assays (knockout/ silencing/ inhibitor/ agonist)	Assessed the expression of more than two SARS-CoV-2 viral entry genes or different acetylcholine receptors	Use of multiple assays that measure cytotoxicity in cells	Assessed, oxidative stress and DNA damage and cell senescence markers/ infection. Confirmed with histology / BAL wash	WT SARS-CoV-2 infection
**MEDIUM (2)**	Primary human epithelial cells at ALI for less than 14 days OR Primary non-human epithelial cells at ALI OR unstated amount of time OR human epithelial cell lines for 14+ days	Hamster Animal Model OR tissues	Direct condensate application onto an ALI culture	Intratracheal	Chosen dosage not explained but appropriate controls or lack of human related dose	Protein (or immunostaining) and gene expression analysis performed	Assessed the expression of 1 or 2 of ACE2, TMPRSS2 or different acetylcholine receptors	Use of cell viability assay including oxidative stress markers OR DNA damage OR barrier integrity OR infected cells OR histology	Assessed oxidative stress markers OR DNA damage OR cell senescence confirmed with histology OR Infection	SARS-CoV-2 Pseudovirus infection OR SARS-CoV-2 infected samples
**LOW (1)**	Non-ALI cultures OR cell lines at ALI for less than 14 days OR non-differentiated microfluidics	Other Animal Model OR tissues	Direct condensate application into a submerged culture OR a biopsy sample from a smoker/ SARS-CoV-2 positive patient OR basal media in ALI/ microfluidics	Whole body exposure OR intraperitoneally OR unknown	Chosen dosage not explained with a lack of controls OR Chosen dosage related to human exposure with a lack of controls OR Smoker/non-smoker exposure biopsy	A protein or gene expression assay used	No SARS-CoV-2 or acetylcholine receptor genes of interest expression assessed	Did not assess cytotoxicity OR barrier integrity OR did not state methods	Damage not confirmed with histology or BAL	No infection

Abbreviations: ALI = Air-liquid interface; NHP = Non-human primates; BAL = Bronchoalveolar lavage; WT = Wild type.

Supplementary information 4: Quality assessment summary of all included studies. Where multiple scores were relevant for a single study, the highest applicable score is stated. Overall scores were classified by the average score. Values between: 1.00 and 1.25 were rated as VL = very low; 1.26 and 1.50 as L = Low; 1.51 and 2.00 as M = Medium; 2.01 and 3.00 as H = High.

Paper reference	Cell model		Route of exposure		Dosage	Gene and protein expression		Cytotoxicity		SARS-CoV-2 challenge	Overall score	
	In vitro	In vivo	In vitro	In vivo		Assays	Genes	In vitro	In vivo		Average	Rating
Agraval, H., et al., 2022	1		1		2	2	1	2		1	1.4	L
Akihiro Tsutsumi, 2020	2	1	1	3	3	3	1	2	2	1	1.9	M
Albano, G.D., et al., 2018	1		1		2	2	1	1		1	1.3	L
Aliee, H., et al., 2020	1		1		1	2	3	1		1	1.4	L
Al-Wadei, H.A.N., et al., 2010	1		1		3	3	2	1		1	1.7	M
Andrault, P., et al., 2019	1		1		3	3	1	3		1	1.9	M
Arredondo, J., et al., 2006	1		1		1	3	3	2		1	1.7	M
Basil, M. et al., 2022.	1	1	1	1	2	2	1	1	1	1	1.2	VL
Blank, U., et al., 1997	1		1		2	2	1	1		1	1.3	L
Cai, G., et al., 2020	1		1		1	1	3	1		1	1.3	L
Carlier, F. et al., 2021	3		1		1	2	1	2		1	1.6	M
Carlisle, D.L., et al., 2004	1		1		3	3	3	1		1	1.9	M
Chakladar, J., et al., 2020	1		1		1	1	3	1		1	1.3	L
Chen, P., et al., 2021.	1		1		2	3	1	1		1	1.4	L
Chen, Q., et al., 2021		1		3	2	2	1	1	2	3	1.9	M
Chen, Q., et al., 2022	3		2		2	2	3	2		3	2.4	H
Christian Ginzkey, 2012	1		1		3	3	2	3		1	2.0	M
Chu, M., et al., 2005	1		1		2	3	2	1		1	1.6	M
Chung, S., et al., 2015	1	1	1	1	2	3	1	2	2	1	1.5	L

S4 continued…

Paper reference	Cell model		Route of exposure		Dosage	Gene and protein expression		Cytotoxicity		SARS-CoV-2 challenge	Overall score	
	In vitro	In vivo	In vitro	In vivo		Assays	Genes	In vitro	In vivo		Average	Rating
Chung, S., et al., 2019	3	1	3	3	2	3	1	1	1	1	1.9	M
Cortijo, J., et al., 2011	3		1		2	3	1	2		1	1.9	M
Daniell, H., et al., 2022	1		1		1	2	2	1		3	1.6	M
Di Vincenzo, S. et al., 2022	3		3		1	2	1	2		1	1.9	M
Downs, C., et al., 2011	1		1		3	2	1	3		1	1.7	M
Du, H., et al., 2012	1		3		3	2	1	3		1	2.0	M
Ebrahimpour, A., et al., 2019	1		1		2	1	2	1		1	1.3	L
Eurlings, I., et al., 2014	1	1	1	1	3	3	1	3	2	1	1.7	M
Foronjy, R., et al., 2016	3	1	3	1	3	3	1	2	3	1	2.1	H
Fu, X., et al., 2009	1		1		3	3	3	1		1	1.9	M
Gahring, L.C., et al., 2017		1		1	2	3	1		2	1	1.6	M
Gao, H., et al., 2020	1		1		3	2	1	2		1	1.6	M
Gebel, S., et al., 2006		1		3	3	3	1		2	1	2.0	M
Geraghty, P., et al., 2014.	1	1	1	1	2	3	1	2	2	1	1.5	L
Ghosh, A., et al., 2021	2		3		2	2	3	2		2	2.3	H
Golovatch, P., et al., 2009		1		1	2	2	1		2	1	1.4	L
Gundavarapu, S., et al., 2012	2		2		3	3	3	2		1	2.3	H
Guo, J., et al., 2005	1		1		1	3	1	2		1	1.4	L
Haslbauer, J., et al., 2022	1		1		1	3	3	1		3	1.9	M
Hirschi-Budge, K. et al., 2022		1		3	2	3	1		1	1	1.7	M
Ho, Y., et al., 2005	1	1	1	1	3	3	3	2	2	1	1.8	M

S4 continued…

Paper reference	Cell model		Route of exposure		Dosage	Gene and protein expression		Cytotoxicity		SARS-CoV-2 challenge	Overall score	
	In vitro	In vivo	In vitro	In vivo		Assays	Genes	In vitro	In vivo		Average	Rating
Hou, W., et al., 2020	2		2		3	3	1	2		1	2.0	M
Hudlikar, R., et al., 2022.		1		1	1	3	1		2	1	1.4	L
Hung, Y., et al., 2016		1		1	2	3	2		3	1	1.9	M
Irie, H., et al., 2022		1		3	1	3	1		3	1	1.9	M
Izzotti, A., et al., 2010		1		1	1	2	1		1	1	1.1	VL
Jiang, J.-X., 2017	1	1	1	1	3	3	1	1	1	1	1.4	L
Kasper, B., et al., 2022	3		1		1	2	3	1		1	1.7	M
Lallai, V., Manca, L. and Fowler, C., 2021		1		1	2	2	3		1	1	1.6	M
Lam, D., et al., 2016	1		1		3	2	3	2		1	1.9	M
Latha, M., et al., 1991		1		1	1	1	1		1	1	1.0	VL
Lee, A., et al., 2020	1		1		1	1	2	1		1	1.1	VL
Lee, H. and J. Kim, 2013	1		1		3	1	1	2		1	1.4	L
Lee, I., et al., 2020	1	1	1	1	1	2	3	1	1	2	1.4	L
Lemjabbar, H., et al., 2003	1	1	1	3	2	3	1	2	2	1	1.7	M
Leung, J., et al., 2020	1		1		1	2	3	1		1	1.4	L
Li, D., et al., 2022		1		1	2	3	2		2	2	1.9	M
Li, E., et al., 2014	1		1		3	3	1	2		1	1.7	M
Li, F., et al., 2012		1		1	3	3	1		2	1	1.7	M
Li, F., et al., 2021	1		1		2	3	2	3		2	2.0	H
Li, G., et al., 2020	1		1		1	1	2	1		1	1.1	VL
Li, H., et al., 2021	3		2		2	3	1	3		1	2.1	H

S4 continued…

Paper reference	Cell model		Route of exposure		Dosage	Gene and protein expression		Cytotoxicity		SARS-CoV-2 challenge	Overall score	
	In vitro	In vivo	In vitro	In vivo		Assays	Genes	In vitro	In vivo		Average	Rating
Li, Q., et al., 2010	1		1		3	3	1	2		1	1.7	M
Li, Q., et al., 2011	1		1		2	3	3	1		1	1.7	M
Li, Q., et al., 2012	1		1		2	3	3	2		1	1.9	M
Li, W., et al., 2021	1		1		1	2	2	1		1	1.3	L
Lin, C., et al., 2021	1		1		1	2	3	2		1	1.6	M
Liu, A., et al., 2021	1	1	1	1	2	3	2	3	3	1	1.8	M
Lupacchini, L., et al., 2020	1		1		2	3	3	3		1	2.0	M
Lv, J., et al., 2021	1	3	1	2	1	3	2	1	2	3	1.9	M
Ma, L., et al., 2020	1	1	1	1	2	3	1	1	1	1	1.3	L
Maehira, F., et al., 1999		1		1	2	2	1		1	1	1.3	L
Mao, Y. and Feng, H., 2022	1		1		3	3	1	2		1	1.7	M
Marshall, K., et al., 2020		1		1	3	2	1		2	1	1.6	M
Martinez-Garcia, E., et al., 2008	1		1		3	3	3	1		1	1.9	M
Masso-Silva, J., et al., 2021		1		1	3	1	2		1	1	1.4	L
McAlinden, K., et al., 2021	1		1		3	2	2	3		1	1.9	M
Mebratu, Y., et al., 2011	2	1	1	1	3	3	1	2	2	1	1.7	M
Mishra, R., et al., 2016	2	1	2	1	3	3	1	2	1	1	1.7	M
Monzon, M., et al., 2011.	3		1		1	3	1	2		1	1.7	M
Nakayama, T., et al., 2021	3		2		3	2	3	3		3	2.7	H
Nath, S., et al., 2019		1		1	2	3	1		1	1	1.4	L
O'Donnell, R. et al., 2004	1		1		1	2	1	1		1	1.1	VL

S4 continued…

Paper reference	Cell model		Route of exposure		Dosage	Gene and protein expression		Cytotoxicity		SARS-CoV-2 challenge	Overall score	
	In vitro	In vivo	In vitro	In vivo		Assays	Genes	In vitro	In vivo		Average	Rating
Ogawa, F., et al., 2019	3		2		1	3	1	1		1	1.7	M
Osborne, J. et al., 2014	1		1		3	3	3	1		1	1.9	M
Peng, Y., et al., 2022	1		1		1	1	3	1		1	1.3	L
Phandthong, R., et al., 2022	1		3		3	2	2	2		2	2.1	H
Podowski, M., e al., 2012		1		1	1	3	1		2	1	1.4	L
Polk, W., 2012	1		1		3	3	1	3		1	1.9	M
Polosukhin, V., et al., 2011	2		1		1	3	1	2		1	1.6	M
Profita, M., et al., 2011	1		1		2	3	1	1		1	1.4	L
Qiao, Y., et al., 2021	1		1		1	3	2	2		3	1.9	M
Qixin Wang, 2020		1		1	2	3	3		2	1	1.9	M
Rangasamy, T., et al., 2009		1		1	2	3	1		2	1	1.6	M
Rayner, R., et al., 2022	3		2		3	1	1	2		1	1.9	M
Russo, P., et al., 2020	1		1		2	2	2	1		1	1.4	L
Sarkar, P. and Hayes, B., 2007	1		1		3	3	1	1		1	1.6	M
Shaykhiev, R., et al., 2013	3		2		2	2	1	2		1	1.9	M
Shen, H., et al., 2022	1	1	1	1	2	3	1	1	3	1	1.5	M
Sompel, K., et al., 2022	1	1	1	1	2	3	1	1	1	1	1.3	L
Staudt, M., et al., 2018	1		1		1	1	1	1		1	1.0	VL
Tan, L., et al., 2018	1	1	1	1	2	3	1	1	1	1	1.3	L
Tang, X., et al., 2021	1	1	1	1	3	3	1	3	3	1	1.8	M
Tomchaney, M., et al., 2020	2	1	3	1	2	2	2	2	1	3	1.9	M

S4 continued…

Paper reference	Cell model		Route of exposure		Dosage	Gene and protein expression		Cytotoxicity		SARS-CoV-2 challenge	Overall score	
	In vitro	In vivo	In vitro	In vivo		Assays	Genes	In vitro	In vivo		Average	Rating
Van der Toorn, M., et al., 2017	1		1		3	3	1	2		1	1.7	M
Veljkovic, E., et al., 2011	1		1		2	3	2	2		1	1.7	M
Vivarelli, F., et al., 2021		1		1	2	2	1		3	1	1.6	M
Voinsky, I. and D. Gurwitz, 2020	1		1		1	1	3	1		1	1.3	L
Wang, G., et al., 2021	1	1	1	1	3	3	3	1	2	2	1.8	M
Wang, Q., et al., 2014	1		1		2	3	1	2		1	1.6	M
Wang, Y., et al., 2001	1		1		1	3	2	1		1	1.4	L
Wark, P., et al., 2021	2		1		1	2	3	1		1	1.6	M
West, K., et al., 2003	1		1		3	3	3	3		1	2.1	H
Wick, K. et al., 2022	2		3		2	2	1	3		1	2.0	H
Wohnhaas, C., et al., 2021	3		3		3	2	3	2		1	2.4	H
Yang, J., et al., 2017	3		1		1	3	1	1		1	1.6	M
Yang, J., et al., 2021	1	1	1		1	3	2	1		2	1.5	L
Yu, H., et al., 2011	1		1		3	3	1	3		1	1.9	M
Yue, X., et al., 1992		1		1	1	1	2		1	1	1.1	VL
Zhang, L., et al., 2013	3		2		3	3	1	1		1	2.0	H
Zhang, L., et al., 2013	3		2		2	3	1	1		1	1.9	M
Zhang, L., et al., 2014	2		1		1	3	1	1		1	1.4	L
Zhang, L., et al., 2016	1		1		1	3	1	1		1	1.3	L
Zhang, Y., et al., 2021.	1	1	1	2	3	3	1	3	3	1	1.9	M
Zhao, Y., et al., 2013	1		1		3	3	1	3		1	1.9	M

S4 continued…

Paper reference	Cell model		Route of exposure		Dosage	Gene and protein expression		Cytotoxicity		SARS-CoV-2 challenge	Overall score	
	In vitro	In vivo	In vitro	In vivo		Assays	Genes	In vitro	In vivo		Average	Rating
Zia, S., et al., 1997	1	1	1	1	2	2	3	2	2	1	1.6	M

Supplementary information 5: Confidence scoring of genes extracted from all included studies after converting non-human GOI to their human homologues. Confidence scores combined study frequency, (the number of studies investigating the gene of interest (GOI)), consistency (the overall level of change of the GOI across all studies) and quality (the overall quality score of the studies from which each GOI was extracted). The higher the positive confidence score, the more robust the evidence for upregulation and the greater the negative confidence score, the more robust the evidence for downregulation.

Human translated gene (NCBI)	Gene ID	Cigarette smoke exposure		E-cigarette exposure (No nicotine)		E-cigarette exposure (Nicotine)		Nicotine alone exposure		SARS-CoV-2 exposure	
		mRNA	Protein	mRNA	Protein	mRNA	Protein	mRNA	Protein	mRNA	Protein
ABCB6	10058	1.5				1.5					
ACE	1636	0.5	1.5								
ACE2	59272	12.5	5		-1.5	2	6.5	1	4	6.5	
ACTA2	59	1.0	4								
ACTL6B	51412	1.5									
ADAM17	6868	1.0	0								
ADAM19	8728	1.5									
ADAM8	101	1.5				-1.5					
ADAMTS12	81792	1.5									
ADAMTS3	9508	1.5									
ADAMTSL4	54507	1.0	1								
ADH7	131	5.0									
ADRB2	154		1								
AGER	177	1.5	1.5								
AGT	183					-1					
AGTR1	185	1.5									-1.5
AHR	196	1.5	1.5				1.5			1.5	
AIMP1	9255							1			
AJUBA	84962					0.5					
AKAP13	11214					1.5					
AKR1B10	57016	2.0									
AKR1C1	1645	3.5									
AKR1C2	1646	4.5				2					
AKR1C3	8644	2.0									
AKR1D1	6718	1.0									
AKT1	207	2.5	3.5					2	3		-2
ALDH1A3	220	2.0									
ALDH3A1	218	6.5	2								
AMOTL2	51421					0.5					
APOE	348	-1.5									
AR	367	1.0									
ARF6	382			1.5							
ARNT	405	1.5									
ARX	170302	-1.5									
ATXN1	6310	-1									
AURKA	6790		-1								
AVPR1A	552	-1.5									

S5 continued…

Human translated gene (NCBI)	Gene ID	Cigarette smoke exposure		E-cigarette exposure (No nicotine)		E-cigarette exposure (Nicotine)		Nicotine alone exposure		SARS-CoV-2 exposure	
		mRNA	Protein	mRNA	Protein	mRNA	Protein	mRNA	Protein	mRNA	Protein
BATF3	55509			0.5							
BAX	581	1.0									2
BCL2	596	2.5	1.5								-2
BCL2L1	598	1.5	1.5			1.5	1.5				
BEST3	144453					-0.5					
BIK	638	-3									
BIRC5	332	2		-1.5							
BMAL1	406	-1.5									
BPIFA2	140683	2.0									
BSG	682	1.5	2.5								
BTG2	7832					-1.5					
C3	718	2.0									
C4A	720			-1.5							
CA9	768	3.0									
CALML5	51806			-0.5							
CASP3	836	1.0	-0.5								
CASP7	840					-1.5					
CCL11	6356									1.5	
CCL2	6347	1.0	1.5			-2				1.5	
CCL20	6364	-1.5									
CCL3	6348	1.5									
CCL5	6352	-1.5									
CCL7	6354									1.5	
CCN2	1490		1								
CCND1	595	1.5	6.5						2.5		
CCNJ	54619	-1.5									
CCR2	729230		1.5			-1					
CCR7	1236			1.5							
CD151	977					1.5					
CD177	57126					-1					
CD40	958					-1					
CD44	960	3.0									
CD68	968		1								
CD74	972	1.5									
CDC42	998	2.0									
CDH1	999	-17.5	-16			-1.5	-1.5	-1.5			
CDH2	1000	1.5	3								
CDK9	1025	-1									
CDKN1A	1026	1.5	3								
CDKN2A	1029	1.5	1.5								
CDKN2C	1031		-1								
CEACAM5	1048	1.5									
CELSR1	9620	-1.5									

S5 continued…

Human translated gene (NCBI)	Gene ID	Cigarette smoke exposure		E-cigarette exposure (No nicotine)		E-cigarette exposure (Nicotine)		Nicotine alone exposure		SARS-CoV-2 exposure	
		mRNA	Protein	mRNA	Protein	mRNA	Protein	mRNA	Protein	mRNA	Protein
CES3	23491	1.0									
CFB	629	1.5									
CFTR	1080					-2					
CHAT	1103	2.0	3								
CHIT1	1118										
CHRM2	1129	2.0	2								
CHRM3	1131	2.5	2.5								
CHRNA1	1134	1.5	1.5					1.5	1.5		
CHRNA3	1136	1.5						5			
CHRNA4	1137							3.5			
CHRNA5	1138	1.5	1.5			-1.5		6	3		
CHRNA6	8973	-1.5						1.5			
CHRNA7	1139	1.5	1.5					10.5	4.5		
CHRNB2	1141	-1.5	1.5					1.5			
CHRNB4	1143	-1.5						1.5			
CHRNG	1146	-1.5	1.5								
CLCA1	1179	-1.5									
CLDN1	9076	-2		1.5							
CLDN10	9071	1.0									
CLDN3	1365	-1.5									
CLDN7	1366	2.0									
CLDN8	9073	2.0									
CLEC4M	10332	1.5	1.5								
CNN2	1265					-1.5					
COL12A1	1303					1.5					
COL1A1	1277	1.5	0.5				-1.5				
COL1A2	1278			-1.5		-1.5					
COL4A1	1282			-1.5		-1.5					
COX2	4513	5.0									
COX4I1	1327	-1.5									
CPT1A	1374		1.5								
CRIP1	1396			1.5		1.5					
CSF2	1437	3.0									
CSF3	1440	1.5									
CST6	1474					0.5					
CTNNB1	1499	-0.5	-2								
CTNND1	1500	2.0	-1.5								
CTSB	1508	1.5									
CTSD	1509	2.0									
CTSE	1510	1.0									
CTSG	1511	1.0									
CTSK	1513	2.5	1								
CTSL	1514	-1									

S5 continued…

Human translated gene (NCBI)	Gene ID	Cigarette smoke exposure		E-cigarette exposure (No nicotine)		E-cigarette exposure (Nicotine)		Nicotine alone exposure		SARS-CoV-2 exposure	
		mRNA	Protein	mRNA	Protein	mRNA	Protein	mRNA	Protein	mRNA	Protein
CTSS	1520	2.5	2.5	1.5							
CX3CL1	6376	1.0									
CXCL1	2919	2.0	1.5								
CXCL10	3627	-3								1.5	
CXCL17	284340	2.0									
CXCL2	2920					-2					
CXCL5	6374	0.5									
CXCL8	3576	12.5	3.5					1.5	1.5		2
CYBC1	79415		2								
CYGB	114757	-1.5									
CYP1A1	1543	13.0	1.5			2	1.5				
CYP1B1	1545	6.5				3.5					
CYP26A1	1592	1.5									
CYP2A13	1553	1.5	1.5				1.5				
CYP2A6	1548	1.5	1.5				1.5				
CYP2A7	1549	1.5									
CYP2B6	1555	1.5									
CYP2C19	1557	2.0									
CYP2C9	1559	2.0									
CYP2E1	1571	1.5									
CYP2F1	1572	-3.5									
CYP2J2	1573	1.0									
CYP3A4	1576	1.0				1.5					
CYP3A43	64816	1.0									
CYP3A5	1577	1.0									
CYP4F3	4051	1.5									
DBP	1628	1.5									
DEFB4B	1E+08	-1.5									
DLGAP5	9787			-0.5							
DMBT1	1755	2.0									
DNAI1	27019	-3									
DNASE1L2	1775			-0.5							
EGF	1950	0.0	1.5								
EGFR	1956	2.0	6.5						7		
EGR1	1958					-2					
EGR3	1960	1.0									
EIF4EBP1	1978							2			
ELK1	2002	-0.5									
ELN	2006		-1								
EN1	2019	-1.5									
EPPK1	83481	-1.5									
ERBB2	2064	-1.5	3								
ERBB3	2065	1.5	5								

S5 continued…

Human translated gene (NCBI)	Gene ID	Cigarette smoke exposure		E-cigarette exposure (No nicotine)		E-cigarette exposure (Nicotine)		Nicotine alone exposure		SARS-CoV-2 exposure	
		mRNA	Protein	mRNA	Protein	mRNA	Protein	mRNA	Protein	mRNA	Protein
ERBB4	2066	1.5									
ERCC6	2074	1.5									
ERN1	2081		1.5								
EVA1A	84141	-1.5									
FAM114A1	92689	1.0									
FCGRT	2217					-1					
FGFR3	2261	1.0	3								
FKBP8	23770	2.0									
FMO3	2328	2.0									
FN1	2335	1.5	3		-1.5				2		
FOLR1	2348	-1.5									
FOS	2352	-1.0				-2					
FOSB	2354	1.0									
FOSL1	8061	1.0									
FOXA2	3170	-1.5									
FOXA3	3171	1.5									
FOXJ1	2302	-3									
FOXO1	2308							2			
FRAT1	10023	-1									
FTH1	2495	2.0	-1.5								
FTL	2512	3.5									
FURIN	5045	2.0									
FZD9	8326	-1									
GATA3	2625	1.5									
GATA6	2627			1.5							
GCLC	2729	3.5									
GCLM	2730	1.5									
GEM	2669					-1.5					
GPX1	2876	3.5									
GPX2	2877	8.0									
GPX4	2879		-1.5								
GRHL3	57822			0.5							
GSK3B	2932							2			
GSS	2937		-1.5								
GSTP1	2950	2.0									
GUSB	2990	0.5									
H2AX	3014		3								
HDC	3067			-1.5							
HERPUD1	9709	1.5	1.5								
HES1	3280	-1.5									
HIF1A	3091	4.5	1.5								
HLA-DMA	3108		1.5								
HLA-DQA1	3117			1.5							

S5 continued…

Human translated gene (NCBI)	Gene ID	Cigarette smoke exposure		E-cigarette exposure (No nicotine)		E-cigarette exposure (Nicotine)		Nicotine alone exposure		SARS-CoV-2 exposure	
		mRNA	Protein	mRNA	Protein	mRNA	Protein	mRNA	Protein	mRNA	Protein
HLA-E	3133					-1.5					
HLA-F	3134					-1.5					
HLA-G	3135					-1.5					
HMGCS2	3158	-1.5									
HRAS	3265	3.0	3						2.5		
HRG	3273	1.0									
HSP90AA1	3320	1.5									
HSPA5	3309	1.5	3								
ICOSLG	23308			1.5							
IFNB1	3456		2								
IFNG	3458	1.5				-1				1.5	
IFNL3	282617									1.5	
IL10	3586	1.5								0.5	
IL12A	3592	1.5									
IL13	3596	1.5									
IL17A	3605									1.5	
IL17B	27190							1			
IL17RA	23765	1.0									
IL1A	3552	3				-1				2	
IL1B	3553	4.5						1			
IL1R2	7850	1.5		-1.5		-1.5					
IL2	3558	1.5				-1				1.5	
IL21	59067							1			
IL22	50616									1.5	
IL24	11009		1.5								
IL2RB	3560							1			
IL36A	27179	1.5									
IL5	3567									1.5	
IL6	3569	13.5	7			-1		1.5	1.5	2	2
ISG20	3669			0.5							
ITGAM	3684		1								
ITGAV	3685	-1									
IV	8114		1.5								
IVL	3713	1.5									
JAG1	182	-1.5									
JAK1	3716					-1					
JPH2	57158	1.0									
JUN	3725					-2					
KAT5	10524	1.5									
KCNK15	60598			-0.5							
KLHL13	90293	-1.5									
KRAS	3845	4.0	1.5								
KRT13	3860	1.5									

S5 continued…

Human translated gene (NCBI)	Gene ID	Cigarette smoke exposure		E-cigarette exposure (No nicotine)		E-cigarette exposure (Nicotine)		Nicotine alone exposure		SARS-CoV-2 exposure	
		mRNA	Protein	mRNA	Protein	mRNA	Protein	mRNA	Protein	mRNA	Protein
KRT14	3861	1.5									
KRT18	3875	-1.5									
KRT5	3852	-1.5									
KRT6A	3853	1.5									
KRT7	3855	-1.5									
KRT83	3889					1					
KRT9	3857	-1.5									
LAMP1	3916	-1.5									
LAMP3	27074	0.5									
LATS2	26524					0.5					
LCN2	3934	2.0									
LDLR	3949					-1.5					
LGALS7B	653499			0.5							
LMNA	4000		-1.5								
LTB4R	1241	1.0	1								
LTB4R2	56413			0.5							
LTF	4057	-1.5									
LY6G6D	58530			-1.5							
LY6H	4062	1.5									
MAGEA6	4105	1.5				1.5					
MAP1B	4131	1.5									
MAP1LC3A	84557	0									
MAP1LC3B	81631										2
MAP2K2	5605		1								
MAP2K3	5606					1.5					
MAP2K6	5608	1.5									
MAP3K1	4214	1.5									
MAP3K4	4216		1.5								
MAP7	9053	-1.5									
MAPK1	5594	7.5	20.5			-2		1			
MAPK14	1432	5.0	6					1.5	2		
MAPK3	5595	1.5						1			
MAPK8	5599	1.5	8								
MAPK9	5601	1.0	1								
MARCKSL1	65108			-1.5							
ME1	4199	1.0									
MGMT	4255	-2									
MGP	4256	-1.5									
MGST1	4257	2.0									
MKI67	4288								2		
MMP1	4312	3.0									
MMP12	4321	3.0					1.5				
MMP14	4323	-1.5									

S5 continued…

Human translated gene (NCBI)	Gene ID	Cigarette smoke exposure		E-cigarette exposure (No nicotine)		E-cigarette exposure (Nicotine)		Nicotine alone exposure		SARS-CoV-2 exposure	
		mRNA	Protein	mRNA	Protein	mRNA	Protein	mRNA	Protein	mRNA	Protein
MMP2	4313	1.5	0		1.5		1.5				
MMP3	4314	1.5									
MMP7	4316	-1.5									
MMP8	4317			-1.5	-1.5	1.5					
MMP9	4318	4.0	0.5	-1.5	-1.5		1.5				
MPHOSPH8	54737	-1									
MS4A1	931			1.5							
MSS51	118490	1.5									
MT1A	4489	2.0									
MT1X	4501	2.0				-0.5					
MT2A	4502					-0.5					
MTOR	2475		1.5								-2
MUC1	4582	0.5	0.5								
MUC20	200958	-1.5									
MUC5AC	4586	10.0	8.5					2	2		
MUC5B	727897	-1.5	1.5								
MUCL1	118430	1.5									
MYB	4602	-1.5									
MYBL1	4603	2.0									
MYC	4609		2								
MYD88	4615	1.5	1.5								
MYL5	4636					-0.5					
NCAM1	4684							1.5			
NECTIN1	5818					-1.5					
NEFM	4741							1.5			
NEURL3	93082					-0.5					
NEUROD1	4760							1.5	1.5		
NFE2	4778		-3								
NFE2L2	4780	0.0	1.5				1.5				
NFKB1	4790	5.5	8.5			-1	3	1			
NFKBIA	4792		6								
NFKBIB	4793					-1					
NKRF	55922	1.0									
NLRP3	114548	-1.5									
NOD1	10392					-2					
NOS2	4843	1.5									
NOTCH1	4851	-1.5									
NOX4	50507		1								
NPAS2	4862	-1.5									
NPR3	4883	-1.5									
NQO1	1728	8.0	2								
NR1D1	9572	3.0				-0.5					
NR1D2	9975	2.0									

S5 continued…

Human translated gene (NCBI)	Gene ID	Cigarette smoke exposure		E-cigarette exposure (No nicotine)		E-cigarette exposure (Nicotine)		Nicotine alone exposure		SARS-CoV-2 exposure	
		mRNA	Protein	mRNA	Protein	mRNA	Protein	mRNA	Protein	mRNA	Protein
NR3C2	4306					-1					
NRARP	441478	-1									
NRG1	3064	1.5	1.5								
NRP1	8829	-1.5									
OCLN	1E+08	-3.5	-1.5								
OGG1	4968		2.5								
OSGIN1	29948	1.5									
PAK3	5063	1.5									
PARD3	56288	-1.5									
PARD6A	50855	-1.5									
PARG	8505	0.5									
PARP1	142	1.5									
PAX3	5077							1.5			
PCNA	5111	1.5									
PDK2	5164	-1									
PEBP1	5037		1								
PGM2L1	283209					2					
PIGR	5284	1.5									
PIK3CA	5290		1.5			-1			4.5		
PIR	8544	1.0									
PLAUR	5329	1.5	1.5								
PLXDC1	57125			-0.5							
PPARG	5468	-2									
PPP1R16B	26051	1.0									
PRDX1	5052	2.0									
PRKCA	5578	-1				1.5		1.5	1		
PRKCI	5584	0.0									
PRNP	5621	-1.5									
PSMB9	5698					-1.5					
PTEN	5728	-1.5									
PTPN11	5781	1.5	1.5								
RAC1	5879	3.0	1								
RAF1	5894	1.0	1.5								
RARRES2	5919	2.0									
RASL11A	387496	-1.5									
RASSF1	11186	-2									
RELA	5970	2.0	3						-1.5		
RETNLB	84666	-1.5									
RNASE1	6035	2.0									
RND3	390					0.5					
ROS1	6098	1.5							1		
RPS6	6194							1			
RPS6KB1	6198							2			

S5 continued…

Human translated gene (NCBI)	Gene ID	Cigarette smoke exposure		E-cigarette exposure (No nicotine)		E-cigarette exposure (Nicotine)		Nicotine alone exposure		SARS-CoV-2 exposure	
		mRNA	Protein	mRNA	Protein	mRNA	Protein	mRNA	Protein	mRNA	Protein
S100A10	6281	1.0									
S100A8	6279	2.0		-1.5							
S100A9	6280	2.0									
SAA1	6288	2.0									
SAA2	6289	2.0									
SCGB1A1	7356	-1.5	-1.5								
SCGB3A2	117156	-1									
SDR39U1	56948	-1									
SERPINB2	5055					0.5					
SERPINE1	5054	1.5					1.5				
SFN	2810	1.5									
SFTPB	6439	-1.5									
SGK1	6446					-0.5					
SGPP1	81537					1.5					
SIRT1	23411	-1.5									
SKIL	6498					-1.5					
SKP2	6502	1.5	1.5								
SLC38A1	81539	-1.5									
SLC38A2	54407					2					
SLC5A3	6526					2					
SLC5A8	160728			0.5							
SLC7A1	6541					-1					
SLC7A11	23657		-1.5			2					
SLC9A5	6553	1.5				1.5					
SMAD2	4087		1								
SMAD3	4088		1.5								
SMPD3	55512	1.0									
SNAI1	6615	1.5									
SNAI2	6591	4.5	1.5								
SNAI3	333929	-1.5									
SOD1	6647		1.5				1.5				
SOX14	8403	1.0									
SOX9	6662	-1.5									
SPDEF	25803	1.5									
SPP1	6696	-2									
SQSTM1	8878										-2
SRC	6714	3.0	3								
SRPX2	27286	1.0									
SRSF5	6430					-2					
ST6GALNAC 3	256435	1.5									
STAB1	23166					1.5					
STAT1	6772	1.5	1.5			-1					

S5 continued…

Human translated gene (NCBI)	Gene ID	Cigarette smoke exposure		E-cigarette exposure (No nicotine)		E-cigarette exposure (Nicotine)		Nicotine alone exposure		SARS-CoV-2 exposure	
		mRNA	Protein	mRNA	Protein	mRNA	Protein	mRNA	Protein	mRNA	Protein
STAT3	6774		3								
STEAP4	79689	1.5									
TACR1	6869	-1.5									
TACSTD2	4070	2.0	2								
TADA2A	6871	-1									
TALDO1	6888	2.0									
TFRC	7037		1.5								
TGFA	7039	-1.5									
TGFB1	7040	1.0	4			-1					
TGFB2	7042	1.5									
TIMP1	7076	1.5									
TIMP2	7077	-1.5									
TJP1	7082	-1.5						-1.5			-2
TKT	7086	2.0									
TLR4	7099	-1.5									
TLR6	10333					1.5					
TLR9	54106	2.0	2			-1					
TMEM171	134285			-0.5							
TMOD1	7111	-1.5									
TMPRSS2	7113	4.5	1.5		-2		2		2		
TMPRSS3	64699	-1.5									
TMPRSS4	56649	3.0									
TNC	3371			-1.5							
TNF	7124	7.0	3			-1		1	0		2
TNFRSF19	55504	1.5									
TNFRSF1B	7133							1			
TNFRSF4	7293							1			
TNFSF10	8743	-1.5									
TP53	7157	-0.5							-1.5		
TP63	8626	-1.5									
TPM3	7170					1.5					
TRAP1	10131							1.5			
TRAPPC4	51399	-1									
TRIM16	10626	1.0									
TRPC7	57113	-1									
TWIST1	7291	1.5						1			
TWIST2	117581	1.5									
TXK	7294			0.5							
TXN	7295	3.0									
TXNDC16	57544			1.5							
TYRP1	7306		1.5								
UGT1A1	54658	1.0									
UGT2B15	7366	2.0									

S5 continued…

Human translated gene (NCBI)	Gene ID	Cigarette smoke exposure		E-cigarette exposure (No nicotine)		E-cigarette exposure (Nicotine)		Nicotine alone exposure		SARS-CoV-2 exposure	
		mRNA	Protein	mRNA	Protein	mRNA	Protein	mRNA	Protein	mRNA	Protein
UGT2B17	7367	2.0									
UPK1B	7348	1.5									
VASP	7408					-1.5					
VEGFA	7422	3.0	1.5						4		
VIM	7431	9.5	8						2		
VNN1	8876	1.5									
WIF1	11197	-1.5									
XIST	7503	1.0									
XPO1	7514	-1.5	-1.5			-1.5	-1.5				
ZBTB17	7709	-1									
ZEB1	6935	1.5									
ZHX2	22882								1.5		
ZMYND11	10771	-2									
ZNF385B	151126					-2					

References:

1. Agraval H, Sharma JR, Prakash N, Yadav UCS. Fisetin suppresses cigarette smoke extract-induced epithelial to mesenchymal transition of airway epithelial cells through regulating COX-2/MMPs/13-catenin pathway. Chemico-Biological Interactions. 2022;351.
2. Al-Wadei HAN, Al-Wadei MH, Masi T, Schuller HM. Chronic exposure to estrogen and the tobacco carcinogen NNK cooperatively modulates nicotinic receptors in small airway epithelial cells. Lung Cancer. 2010;69(1):33-9.
3. Albano GD, Bonanno A, Moscato M, Anzalone G, Di Sano C, Riccobono L, et al. Crosstalk between mAChRM3 and beta 2AR, via acetylcholine PI3/PKC/PBEP1/Raf-1 MEK1/2/ERK1/2 pathway activation, in human bronchial epithelial cells after long-term cigarette smoke exposure. Life Sciences. 2018;192:99-109.
4. Aliee H, Massip F, Qi C, Stella de Biase M, van Nijnatten JL, Kersten ETG, et al. Determinants of SARS-CoV-2 receptor gene expression in upper and lower airways. medRxiv : the preprint server for health sciences. 2020.
5. Andrault P-M, Schamberger AC, Chazeirat T, Sizaret D, Renault J, Staab-Weijnitz CA, et al. Cigarette smoke induces overexpression of active human cathepsin S in lungs from current smokers with or without COPD. American Journal of Physiology-Lung Cellular and Molecular Physiology. 2019;317(5):L625-L38.
6. Arredondo J, Chernyavsky AI, Grando SA. The nicotinic receptor antagonists abolish pathobiologic effects of tobacco-derived nitrosamines on BEP2D cells. Journal of Cancer Research and Clinical Oncology. 2006;132(10):653-63.
7. Basil MC, Cardenas-Diaz FL, Kathiriya JJ, Morley MP, Carl J, Brumwell AN, et al. Human distal airways contain a multipotent secretory cell that can regenerate alveoli. Nature. 2022;604(7904):120-+.
8. Blank U, Ruckes C, Clauss W, Weber WM. Effects of nicotine on human nasal epithelium: evidence for nicotinic receptors in non-excitable cells. Pflugers Archiv - European Journal of Physiology. 1997;434(5):581-6.
9. Cai G, Bosse Y, Xiao F, Kheradmand F, Amos CI. Tobacco Smoking Increases the Lung Gene Expression of ACE2, the Receptor of SARS-CoV-2. American Journal of Respiratory and Critical Care Medicine. 2020;201(12):1557-9.
10. Carlier FM, Detry B, Lecocq M, Collin AM, Plante-Bordeneuve T, Verleden SE, et al. The memory of airway epithelium damage in smokers and COPD patients. bioRxiv. 2021;22.
11. Carlisle DL, Hopkins TM, Gaither-Davis A, Silhanek MJ, Luketich JD, Christie NA, et al. Nicotine signals through muscle-type and neuronal nicotinic acetylcholine receptors in both human bronchial epithelial cells and airway fibroblasts. Respiratory Research. 2004;5(27-28).
12. Chakladar J, Shende N, Li WT, Rajasekaran M, Chang EY, Ongkeko WM. Smoking-Mediated Upregulation of the Androgen Pathway Leads to Increased SARS-CoV-2 Susceptibility. International Journal of Molecular Sciences. 2020;21(10).
13. Chen P, Jiang P, Chen J, Yang Y, Guo X. XIST promotes apoptosis and the inflammatory response in CSE-stimulated cells via the miR-200c-3p/EGR3 axis. Bmc Pulmonary Medicine. 2021;21(1).
14. Chen Q, Huang X-Y, Tian Y, Fan C, Sun M, Zhou C, et al. The Infection and Pathogenicity of SARS-CoV-2 Variant B.1.351 in hACE2 Mice. Virologica Sinica. 2021;36(5):1232-5.
15. Chen Q, Langenbach S, Li M, Xia YC, Gao X, Gartner MJ, et al. ACE2 Expression in Organotypic Human Airway Epithelial Cultures and Airway Biopsies. Frontiers in Pharmacology. 2022;13.
16. Chu M, Guo JJ, Chen CY. Long-term exposure to nicotine, via Ras pathway, induces cyclin D1 to stimulate G(1) cell cycle transition. Journal of Biological Chemistry. 2005;280(8):6369-79.
17. Chung S, Baumlin N, Dennis JS, Moore R, Salathe SF, Whitney PL, et al. Electronic Cigarette Vapor with Nicotine Causes Airway Mucociliary Dysfunction Preferentially via TRPA1 Receptors. American Journal of Respiratory and Critical Care Medicine. 2019;200(9):1134-45.
18. Chung S, Vu S, Filosto S, Goldkorn T. Src Regulates Cigarette Smoke-Induced Ceramide Generation via Neutral Sphingomyelinase 2 in the Airway Epithelium. American Journal of Respiratory Cell and Molecular Biology. 2015;52(6):738-48.
19. Cortijo J, Mata M, Milara J, Donet E, Gavalda A, Miralpeix M, et al. Aclidinium inhibits cholinergic and tobacco smoke-induced MUC5AC in human airways. European Respiratory Journal. 2011;37(2):244-54.
20. Daniell H, Nair SK, Shi Y, Wang P, Montone KT, Shaw PA, et al. Decrease in Angiotensin-Converting Enzyme activity but not concentration in plasma/lungs in COVID-19 patients offers clues for diagnosis/treatment. Molecular Therapy-Methods & Clinical Development. 2022;26:266-78.
21. Di Vincenzo S, Ninaber DK, Cipollina C, Ferraro M, Hiemstra PS, Pace E. Cigarette Smoke Impairs Airway Epithelial Wound Repair: Role of Modulation of Epithelial-Mesenchymal Transition Processes and Notch-1 Signaling. Antioxidants (Basel, Switzerland). 2022;11(10).

22. Downs CA, Montgomery DW, Merkle CJ. Age-related differences in cigarette smoke extract-induced H2O2 production by lung endothelial cells. Microvascular Research. 2011;82(3):311-7.
23. Du H, Sun J, Chen Z, Nie J, Tong J, Li J. CIGARETTE SMOKE-INDUCED FAILURE OF APOPTOSIS RESULTING IN ENHANCED NEOPLASTIC TRANSFORMATION IN HUMAN BRONCHIAL EPITHELIAL CELLS. Journal of Toxicology and Environmental Health-Part a-Current Issues. 2012;75(12):707-20.
24. Ebrahimpour A, Shrestha S, Bonnen MD, Eissa NT, Raghu G, Ghebre YT. Nicotine Modulates Growth Factors and MicroRNA to Promote Inflammatory and Fibrotic Processes. Journal of Pharmacology and Experimental Therapeutics. 2019;368(2):169-78.
25. Eurlings IMJ, Reynaert NL, van den Beucken T, Gosker HR, de Theije CC, Verhamme FM, et al. Cigarette Smoke Extract Induces a Phenotypic Shift in Epithelial Cells; Involvement of HIF1 alpha in Mesenchymal Transition. Plos One. 2014;9(10).
26. Foronjy RF, Salathe MA, Dabo AJ, Baumlin N, Cummins N, Eden E, et al. TLR9 expression is required for the development of cigarette smoke-induced emphysema in mice. American Journal of Physiology-Lung Cellular and Molecular Physiology. 2016;311(1):L154-L66.
27. Fu XW, Lindstrom J, Spindel ER. Nicotine Activates and Up-Regulates Nicotinic Acetylcholine Receptors in Bronchial Epithelial Cells. American Journal of Respiratory Cell and Molecular Biology. 2009;41(1):93-9.
28. Gahring LC, Myers EJ, Dunn DM, Weise RB, Rogers SW. Lung epithelial response to cigarette smoke and modulation by the nicotinic alpha 7 receptor. Plos One. 2017;12(11).
29. Gao H, Ye G, Lin Y, Chi Y, Dong S. Benzo a pyrene at human blood equivalent level induces human lung epithelial cell invasion and migration via aryl hydrocarbon receptor signaling. Journal of Applied Toxicology. 2020;40(8):1087-98.
30. Gebel S, Gerstmayer B, Kuhl P, Borlak J, Meurrens K, Mueller T. The kinetics of transcriptomic changes induced by cigarette smoke in rat lungs reveals a specific program of defense, inflammation, and circadian clock gene expression. Toxicological Sciences. 2006;93(2):422-31.
31. Geraghty P, Hardigan A, Foronjy RF. Cigarette Smoke Activates the Proto-oncogene c-Src to Promote Airway Inflammation and Lung Tissue Destruction. American Journal of Respiratory Cell and Molecular Biology. 2014;50(3):559-70.
32. Ghosh A, Girish V, Yuan ML, Coakley RD, Wrennall JA, Alexis NE, et al. Combustible and electronic cigarette exposures increase ACE2 activity and SARS-CoV-2 Spike binding. American Journal of Respiratory and Critical Care Medicine. 2022;205(1):129-33.
33. Ginzkey C, Stueber T, Friehs G, Koehler C, Hackenberg S, Richter E, et al. Analysis of nicotine-induced DNA damage in cells of the human respiratory tract. Toxicology Letters. 2012;208(1):23-9.
34. Golovatch P, Mercer BA, Lemaitre V, Wallace A, Foronjy RF, D'Armiento J. ROLE FOR CATHEPSIN K IN EMPHYSEMA IN SMOKE-EXPOSED GUINEA PIGS. Experimental Lung Research. 2009;35(8):631-45.
35. Gundavarapu S, Wilder JA, Mishra NC, Rir-sima-ah J, Langley RJ, Singh SP, et al. Role of nicotinic receptors and acetylcholine in mucous cell metaplasia, hyperplasia, and airway mucus formation in vitro and in vivo. Journal of Allergy and Clinical Immunology. 2012;130(3):770-+.
36. Guo JJ, Chu M, Abbeyquaye T, Chen CY. Persistent nicotine treatment potentiates amplification of the dihydrofolate reductase gene in rat lung epithelial cells as a consequence of Ras activation. Journal of Biological Chemistry. 2005;280(34):30422-31.
37. Haslbauer JD, Stalder A, Zinner C, Bassetti S, Mertz KD, Went P, et al. Immunohistochemical and Transcriptional Analysis of SARS-CoV-2 Entry Factors and Renin-Angiotensin-Aldosterone System Components in Lethal COVID-19. Pathobiology. 2022;89(3):166-77.
38. Hirschi-Budge KM, Tsai KYF, Curtis KL, Davis GS, Theurer BK, Kruyer AMM, et al. RAGE signaling during tobacco smoke-induced lung inflammation and potential therapeutic utility of SAGEs. Bmc Pulmonary Medicine. 2022;22(1).
39. Ho YS, Chen CH, Wang YJ, Pestell RG, Albanese C, Chen RJ, et al. Tobacco-specific carcinogen 4-(methylnitrosamino)-1-(3-pyridyl)-1-butanone (NNK) induces cell proliferation in normal human bronchial epithelial cells through NF kappa B activation and cyclin D1 up-regulation. Toxicology and Applied Pharmacology. 2005;205(2):133-48.
40. Hou W, Hu S, Yong KT, Zhang J, Ma H. Cigarette smoke-induced malignant transformation via STAT3 signalling in pulmonary epithelial cells in a lung-on-a-chip model. Bio-Design and Manufacturing. 2020;3(4):383-95.
41. Hudlikar RR, Sargsyan D, Cheng D, Kuo H-CD, Wu R, Su X, et al. Tobacco carcinogen 4- methyl(nitroso)amino -1-(3-pyridinyl)-1-butanone (NNK) drives metabolic rewiring and epigenetic reprograming in A/J mice lung cancer model and prevention with diallyl sulphide (DAS). Carcinogenesis. 2022;43(2):140-9.

42. Hung Y-H, Hsieh W-Y, Hsieh J-S, Liu F-C, Tsai C-H, Lu L-C, et al. Alternative Roles of STAT3 and MAPK Signaling Pathways in the MMPs Activation and Progression of Lung Injury Induced by Cigarette Smoke Exposure in ACE2 Knockout Mice. International Journal of Biological Sciences. 2016;12(4):454-65.
43. Irie H, Ozaki M, Chubachi S, Hegab AE, Tsutsumi A, Kameyama N, et al. Short-term intermittent cigarette smoke exposure enhances alveolar type 2 cell stemness via fatty acid oxidation. Respiratory Research. 2022;23(1).
44. Izzotti A, Calin GA, Steele VE, Cartiglia C, Longobardi M, Croce CM, et al. Chemoprevention of Cigarette Smoke-Induced Alterations of MicroRNA Expression in Rat Lungs. Cancer Prevention Research. 2010;3(1):62-72.
45. Jiang J-X, Zhang S-J, Shen H-J, Guan Y, Liu Q, Zhao W, et al. Racl signaling regulates cigarette smoke-induced inflammation in the lung via the Erk1/2 MAPK and STAT3 pathways. Biochimica Et Biophysica Acta-Molecular Basis of Disease. 2017;1863(7):1778-88.
46. Kasper B, Yue X, Goldmann T, Guelsen A, Kugler C, Yu X, et al. Air exposure and cell differentiation are essential for investigation of SARS-CoV-2 entry genes in human primary airway epithelial cells in vitro. Frontiers in Medicine. 2022;9.
47. Lallai V, Manca L, Fowler CD. E-cigarette vape and lung ACE2 expression: Implications for coronavirus vulnerability. Environmental Toxicology and Pharmacology. 2021;86.
48. Lam DCL, Luo SY, Fu KH, Lui MMS, Chan KH, Wistuba II, et al. Nicotinic acetylcholine receptor expression in human airway correlates with lung function. American Journal of Physiology - Lung Cellular and Molecular Physiology. 2016;310(3):L232-L9.
49. Latha MS, Vijayammal PL, Kurup PA. CHANGES IN THE GLYCOSAMINOGLYCANS AND GLYCOPROTEINS IN THE TISSUES IN RATS EXPOSED TO CIGARETTE-SMOKE. Atherosclerosis. 1991;86(1):49-54.
50. Lee AC, Chakladar J, Li WT, Chen C, Chang EY, Wang-Rodriguez J, et al. Tobacco, but Not Nicotine and Flavor-Less Electronic Cigarettes, Induces ACE2 and Immune Dysregulation. International Journal of Molecular Sciences. 2020;21(15).
51. Lee HS, Kim J. Cigarette smoke inhibits nasal airway epithelial cell growth and survival. International Forum of Allergy & Rhinology. 2013;3(3):188-92.
52. Lee IT, Nakayama T, Wu C-T, Goltsev Y, Jiang S, Gall PA, et al. ACE2 localizes to the respiratory cilia and is not increased by ACE inhibitors or ARBs. Nature Communications. 2020;11(1).
53. Lemjabbar H, Li DZ, Gallup M, Sidhu S, Drori E, Basbaum C. Tobacco smoke-induced lung cell proliferation mediated by tumor necrosis factor alpha-converting enzyme and amphiregulin. Journal of Biological Chemistry. 2003;278(28):26202-7.
54. Leung JM, Yang CX, Sin DD. COVID-19 and nicotine as a mediator of ACE-2. European Respiratory Journal. 2020;55(6).
55. Li D, Xiong L, Pan G, Wang T, Li R, Zhu L, et al. Molecular imaging on ACE2-dependent transocular infection of coronavirus. Journal of Medical Virology. 2022;94(10):4878-89.
56. Li E, Xu Z, Liu F, Wang H, Wen J, Shao S, et al. Continual Exposure to Cigarette Smoke Extracts Induces Tumor-Like Transformation of Human Nontumor Bronchial Epithelial Cells in a Microfluidic Chip. Journal of Thoracic Oncology. 2014;9(8):1091-100.
57. Li F, Li J, Wang P-H, Yang N, Huang J, Ou J, et al. SARS-CoV-2 spike promotes inflammation and apoptosis through autophagy by ROS-suppressed PI3K/AKT/mTOR signaling. Biochimica Et Biophysica Acta-Molecular Basis of Disease. 2021;1867(12).
58. Li F-f, Shen J, Shen H-j, Zhang X, Cao R, Zhang Y, et al. Shp2 Plays an Important Role in Acute Cigarette Smoke-Mediated Lung Inflammation. Journal of Immunology. 2012;189(6):3159-67.
59. Li G, He X, Zhang L, Ran Q, Wang J, Xiong A, et al. Assessing ACE2 expression patterns in lung tissues in the pathogenesis of COVID-19. Journal of Autoimmunity. 2020;112.
60. Li H, Cui L, Liu Q, Dou S, Wang W, Xie M, et al. Ginsenoside Rb3 Alleviates CSE-induced TROP2 Upregulation through p38 MAPK and NF-kappa B Pathways in Basal Cells. American Journal of Respiratory Cell and Molecular Biology. 2021;64(6):747-59.
61. Li Q, Kolosov VP, Perelman JM, Zhou XD. Src-JNK-dependent pathway in cigarette smoke-induced mucous hypersecretion in airway epithelial cells. Biochemistry (Moscow) Supplement Series A: Membrane and Cell Biology. 2010;4(4):363-73.
62. Li Q, Zhou X, Kolosov VP, Perelman JM. The Expression and Pharmacological Characterization of Nicotinic Acetylcholine Receptor Subunits in HBE16 Airway Epithelial Cells. Cell Biochemistry and Biophysics. 2012;62(3):421-31.

63. Li Q, Zhou XD, Kolosov VP, Perelman JM. Nicotine reduces TNF-alpha expression through a alpha7 nAChR/MyD88/NF-kappaB pathway in HBE16 airway epithelial cells. Cellular Physiology and Biochemistry. 2011;27(5):605-12.
64. Li W, Zheng R, Liang R, Wu B, Wang C, Zhuo L, et al. Effects of Smoking on ACE2 Expression Pattern: Risk and Severity of SARS-CoV-2 Infection. Clinical Laboratory. 2021;67(7):1633-40.
65. Lin C-R, Bahmed K, Simborio H, Hayek H, Bolla S, Marchetti N, et al. Expression of SARS-CoV-2 Entry Factors in Human Alveolar Type II Cells in Aging and Emphysema. Biomedicines. 2021;9(7).
66. Liu A, Zhang X, Li R, Zheng M, Yang S, Dai L, et al. Overexpression of the SARS-CoV-2 receptor ACE2 is induced by cigarette smoke in bronchial and alveolar epithelia. Journal of Pathology. 2021;253(1):17-30.
67. Lupacchini L, Maggi F, Tomino C, De Dominicis C, Mollinari C, Fini M, et al. Nicotine Changes Airway Epithelial Phenotype and May Increase the SARS-COV-2 Infection Severity. Molecules. 2020;26(1).
68. Lv J, Yu P, Wang Z, Deng W, Bao L, Liu J, et al. ACE2 expression is regulated by AhR in SARS-CoV-2-infected macaques. Cellular and Molecular Immunology. 2021;18(5):1308-10.
69. Ma L, Jiang M, Zhao X, Sun J, Pan Q, Chu S. Cigarette and IL-17A synergistically induce bronchial epithelial-mesenchymal transition via activating IL-17R/NF-kappa B signaling. Bmc Pulmonary Medicine. 2020;20(1).
70. Maehira F, Miyagi I, Asato T, Eguchi Y, Takei H, Nakatsuki K, et al. Alterations of protein kinase C, 8-hydroxydeoxyguanosine, and K-ras oncogene in rat lungs exposed to passive smoking. Clinica Chimica Acta. 1999;289(1-2):133-44.
71. Mao Y, Feng H. Vitamin D3 alleviates cigarette smoke extract-mediated epithelial-mesenchymal transition and fibrogenesis by upregulating CC16 expression in bronchial epithelial cells. Experimental and Therapeutic Medicine. 2022;23(5).
72. Marshall K, Liu Z, Olfert IM, Gao W. Chronic electronic cigarette use elicits molecular changes related to pulmonary pathogenesis. Toxicology and Applied Pharmacology. 2020;406.
73. Martinez-Garcia E, Irigoyen M, Anso E, Martinez-Irujo JJ, Rouzaut A. Recurrent exposure to nicotine differentiates human bronchial epithelial cells via epidermal growth factor receptor activation. Toxicology & Applied Pharmacology. 2008;228(3):334-42.
74. Masso-Silva JA, Moshensky A, Shin J, Olay J, Nilaad S, Advani I, et al. Chronic E-Cigarette Aerosol Inhalation Alters the Immune State of the Lungs and Increases ACE2 Expression, Raising Concern for Altered Response and Susceptibility to SARS-CoV-2. Frontiers in Physiology. 2021;12.
75. McAlinden KD, Lu W, Ferdowsi PV, Myers S, Markos J, Larby J, et al. Electronic Cigarette Aerosol Is Cytotoxic and Increases ACE2 Expression on Human Airway Epithelial Cells: Implications for SARS-CoV-2 (COVID-19). Journal of Clinical Medicine. 2021;10(5).
76. Mebratu YA, Schwalm K, Smith KR, Schuyler M, Tesfaigzi Y. Cigarette Smoke Suppresses Bik To Cause Epithelial Cell Hyperplasia and Mucous Cell Metaplasia. American Journal of Respiratory and Critical Care Medicine. 2011;183(11):1531-8.
77. Mishra R, Foster D, Vasu VT, Thaikoottathil JV, Kosmider B, Chu HW, et al. Cigarette Smoke Induces Human Epidermal Receptor 2-Dependent Changes in Epithelial Permeability. American Journal of Respiratory Cell and Molecular Biology. 2016;54(6):853-64.
78. Monzon ME, Forteza RM, Casalino-Matsuda SM. MCP-1/CCR2B-dependent loop upregulates MUC5AC and MUC5B in human airway epithelium. American Journal of Physiology-Lung Cellular and Molecular Physiology. 2011;300(2):L204-L15.
79. Nakayama T, Lee IT, Jiang S, Matter MS, Yan CH, Overdevest JB, et al. Determinants of SARS-CoV-2 entry and replication in airway mucosal tissue and susceptibility in smokers. Cell Reports Medicine. 2021;2(10).
80. Nath S, Doherty DF, Foronjy RF, Ohlmeyer M, Poon J, Dabo AJ, et al. Protein Phosphatase 2A Prevents Cigarette Smoke-Induced Cathepsin S and Loss of Lung Function. American Journal of Respiratory and Critical Care Medicine. 2019;199.
81. O'Donnell RA, Richter A, Ward J, Angco G, Mehta A, Rousseau K, et al. Expression of ErbB receptors and mucins in the airways of long term current smokers. Thorax. 2004;59(12):1032-40.
82. Ogawa F, Walters MS, Shafquat A, O'Beirne SL, Kaner RJ, Mezey JG, et al. Role of KRAS in regulating normal human airway basal cell differentiation. Respiratory Research. 2019;20(1).
83. Osborne JK, Guerra ML, Gonzales JX, McMillan EA, Minna JD, Cobb MH. NeuroD1 mediates nicotine-induced migration and invasion via regulation of the nicotinic acetylcholine receptor subunits in a subset of neural and neuroendocrine carcinomas. Molecular Biology of the Cell. 2014;25(11):1782-92.

84. Peng Y, Zhao-Ni W, Chen SY, Xu AR, Fang ZF, Sun J, et al. Angiotensin-converting enzyme 2 in peripheral lung club cells modulates the susceptibility to SARS-CoV-2 in chronic obstructive pulmonary disease. American Journal of Physiology - Lung Cellular and Molecular Physiology. 2022;322(5):L712-L21.
85. Phandthong R, Wong M, Song A, Martinez T, Talbot P. New Insights into How JUUL Electronic Cigarette Aerosols and Aerosol Constituents Affect SARS-CoV-2 Infection of Human Bronchial Epithelial Cells. bioRxiv : the preprint server for biology. 2022.
86. Podowski M, Calvi C, Metzger S, Misono K, Poonyagariyagorn H, Lopez-Mercado A, et al. Angiotensin receptor blockade attenuates cigarette smoke-induced lung injury and rescues lung architecture in mice. Journal of Clinical Investigation. 2012;122(1):229-40.
87. Polk WW. FH535 Potentiation of Cigarette Smoke Condensate Cytotoxicity is Associated With Changes in beta-Catenin and EGR-1 Signaling. International Journal of Toxicology. 2012;31(4):380-9.
88. Polosukhin VV, Cates JM, Lawson WE, Milstone AP, Matafonov AG, Massion PP, et al. Hypoxia-inducible factor-1 signalling promotes goblet cell hyperplasia in airway epithelium. Journal of Pathology. 2011;224(2):203-11.
89. Profita M, Bonanno A, Montalbano AM, Ferraro M, Siena L, Bruno A, et al. Cigarette smoke extract activates human bronchial epithelial cells affecting non-neuronal cholinergic system signalling in vitro. Life Sciences. 2011;89(1-2):36-43.
90. Qiao Y, Wang X-M, Mannan R, Pitchiaya S, Zhang Y, Wotring JW, et al. Targeting transcriptional regulation of SARS-CoV-2 entry factors ACE2 and TMPRSS2. Proceedings of the National Academy of Sciences of the United States of America. 2021;118(1).
91. Rangasamy T, Misra V, Zhen L, Tankersley CG, Tuder RM, Biswal S. Cigarette smoke-induced emphysema in A/J mice is associated with pulmonary oxidative stress, apoptosis of lung cells, and global alterations in gene expression. American Journal of Physiology-Lung Cellular and Molecular Physiology. 2009;296(6):L888-L900.
92. Rayner RE, Wellmerling J, Makena P, Zhao J, Prasad GL, Cormet-Boyaka E. Transcriptomic Response of Primary Human Bronchial Cells to Repeated Exposures of Cigarette and ENDS Preparations. Cell Biochemistry and Biophysics. 2022;80(1):217-28.
93. Russo P, Bonassi S, Giacconi R, Malavolta M, Tomino C, Maggi F. COVID-19 and smoking: Is nicotine the hidden link? European Respiratory Journal. 2020;55(6) (no pagination).
94. Sarkar P, Hayes BE. Induction of COX-2 by acrolein in rat lung epithelial cells. Molecular and Cellular Biochemistry. 2007;301(1-2):191-9.
95. Shaykhiev R, Zuo W-L, Chao I, Fukui T, Witover B, Brekman A, et al. EGF shifts human airway basal cell fate toward a smoking-associated airway epithelial phenotype. Proceedings of the National Academy of Sciences of the United States of America. 2013;110(29):12102-7.
96. Shen H-j, Jiang J-x, Yao H-y, Zhou H-y, Xie Q-m, Dai H-b, et al. Glycopyrronium bromide regulates cigarette smoke-induced epithelial mesenchymal transition by mediating ACh production. Journal of Molecular Structure. 2022;1266.
97. Sompel K, Dwyer-Nield LD, Smith AJ, Elango AP, Vanderlinden LA, Kopf K, et al. Loss of Frizzled 9 in Lung Cells Alters Epithelial Phenotype and Promotes Premalignant Lesion Development. Frontiers in Oncology. 2022;12.
98. Staudt MR, Salit J, Kaner RJ, Hollmann C, Crystal RG. Altered lung biology of healthy never smokers following acute inhalation of E-cigarettes. Respiratory Research. 2018;19(1) (no pagination).
99. Tan LH, Bahmed K, Lin C-R, Marchetti N, Bolla S, Criner GJ, et al. The cytoprotective role of DJ-1 and p45 NFE2 against human primary alveolar type II cell injury and emphysema. Scientific Reports. 2018;8.
100. Tang X, Li Z, Yu Z, Li J, Zhang J, Wan N, et al. Effect of curcumin on lung epithelial injury and ferroptosis induced by cigarette smoke. Human & Experimental Toxicology. 2021;40(12_SUPPL):S753-S62.
101. Tomchaney M, Contoli M, Mayo J, Baraldo S, Shuaizhi L, Cabel CR, et al. Paradoxical effects of cigarette smoke and COPD on SARS-CoV2 infection and disease. bioRxiv : the preprint server for biology. 2020.
102. Tsutsumi A, Ozaki M, Chubachi S, Irie H, Sato M, Kameyama N, et al. Exposure to Cigarette Smoke Enhances the Sternness of Alveolar Type 2 Cells. American Journal of Respiratory Cell and Molecular Biology. 2020;63(3):293-305.
103. Van der Toorn M, Marescotti D, Johne S, Baumer K, Bornand D, Dulize R, et al. Comparison of the Biological Effects of Long-Term Exposure of Human Bronchial Epithelial Cells to Total Particulate Matter From 3R4F Cigarette Smoke or Aerosol From the Candidate Modified Risk Tobacco Product (cMRTP) THS2.2. International Journal of Toxicology. 2017;36(1):66-7.
104. Veljkovic E, Jiricny J, Menigatti M, Rehrauer H, Han W. Chronic exposure to cigarette smoke condensate in vitro induces epithelial to mesenchymal transition-like changes in human bronchial epithelial cells, BEAS-2B. Toxicology in Vitro. 2011;25(2):446-53.

105. Vivarelli F, Canistro D, Cirillo S, Elias RJ, Granata S, Mussoni M, et al. Unburned Tobacco Cigarette Smoke Alters Rat Ultrastructural Lung Airways and DNA. Nicotine & Tobacco Research. 2021;23(12):2127-34.
106. Voinsky I, Gurwitz D. Smoking andCOVID-19: Similar bronchialACE2andTMPRSS2expression and higherTMPRSS4expression in current versus never smokers. Drug Development Research. 2020;81(8):1073-80.
107. Wang G, Zhao Q, Zhang H, Liang F, Zhang C, Wang J, et al. Degradation of SARS-CoV-2 receptor ACE2 by the E3 ubiquitin ligase Skp2 in lung epithelial cells. Frontiers of Medicine. 2021;15(2):252-63.
108. Wang Q, Wang H, Zhang Y, Xiao W. Activation of uPAR is required for cigarette smoke extract-induced epithelial-mesenchymal transition in lung epithelial cells. Oncology Research. 2014;21(6):295-305.
109. Wang Y, Pereira EFR, Maus ADJ, Ostlie NS, Navaneetham D, Lei S, et al. Human bronchial epithelial and endothelial cells express alpha 7 nicotinic acetylcholine receptors. Molecular Pharmacology. 2001;60(6):1201-9.
110. Wark PAB, Pathinayake PS, Kaiko G, Nichol K, Ali A, Chen L, et al. ACE2 expression is elevated in airway epithelial cells from older and male healthy individuals but reduced in asthma. Respirology. 2021;26(5):442-51.
111. West KA, Brognard J, Clark AS, Linnoila IR, Yang XW, Swain SM, et al. Rapid Akt activation by nicotine and a tobacco carcinogen modulates the phenotype of normal human airway epithelial cells. Journal of Clinical Investigation. 2003;111(1):81-90.
112. Wick KD, Fang X, Maishan M, Matsumoto S, Spottiswoode N, Sarma A, et al. Impact of e-cigarette aerosol on primary human alveolar epithelial type 2 cells. American journal of physiology Lung cellular and molecular physiology. 2022;323(2):L152-L64.
113. Wohnhaas CT, Gindele JA, Kiechle T, Shen Y, Leparc GG, Stierstorfer B, et al. Cigarette Smoke Specifically Affects Small Airway Epithelial Cell Populations and Triggers the Expansion of Inflammatory and Squamous Differentiation Associated Basal Cells. International Journal of Molecular Sciences. 2021;22(14).
114. Yang J, Perez EA, Hou C, Zhang P, Van Scoyk M, Winn RA, et al. Identification of the SARS-CoV-2 Entry Receptor ACE2 as a Direct Target for Transcriptional Repression by Miz1. Frontiers in Immunology. 2021;12.
115. Yang J, Zuo WL, Fukui T, Chao IW, Gomi K, Lee B, et al. Smoking-dependent distal-to-proximal repatterning of the adult human small airway epithelium. American Journal of Respiratory and Critical Care Medicine. 2017;196(3):340-52.
116. Yu H, Li Q, Kolosov VP, Perelman JM, Zhou X. Regulation of Cigarette Smoke-Induced Mucin Expression by Neuregulin1 beta/ErbB3 Signalling in Human Airway Epithelial Cells. Basic & Clinical Pharmacology & Toxicology. 2011;109(1):63-72.
117. Yu XL, Jin XR, Wang DX. Effects of cigarette smoking on the function of metabolizing arachidonic acid and angiotensin I in the isolated perfused rat lungs. Journal of Tongji Medical University = Tong ji yi ke da xue xue bao. 1992;12(4):201-4.
118. Yue X, Basting TM, Flanagan TW, Xu J, Lobell TD, Gilpin NW, et al. Nicotine downregulates the compensatory angiotensin-converting enzyme 2/angiotensin type 2 receptor of the renin–angiotensin system. Annals of the American Thoracic Society. 2018;15(Supplement 2):S126-S7.
119. Zhang L, Gallup M, Zlock L, Basbaum C, Finkbeiner WE, McNamara NA. Cigarette smoke disrupts the integrity of airway adherens junctions through the aberrant interaction of p120-catenin with the cytoplasmic tail of MUC1. Journal of Pathology. 2013;229(1):74-86.
120. Zhang L, Gallup M, Zlock L, Chen YTF, Finkbeiner WE, McNamara NA. Pivotal role of MUC1 glycosylation by cigarette smoke in modulating disruption of airway adherens junctions in vitro. Journal of Pathology. 2014;234(1):60-73.
121. Zhang L, Gallup M, Zlock L, Chen YTF, Finkbeiner WE, McNamara NA. Cigarette Smoke Mediates Nuclear to Cytoplasmic Trafficking of Transcriptional Inhibitor Kaiso through MUC1 and P120-Catenin. American Journal of Pathology. 2016;186(12):3146-59.
122. Zhang L, Gallup M, Zlock L, Finkbeiner WE, McNamara NA. Rac1 and Cdc42 Differentially Modulate Cigarette Smoke Induced Airway Cell Migration through p120-Catenin-Dependent and-Independent Pathways. American Journal of Pathology. 2013;182(6):1986-95.
123. Zhang Y, Huang W, Zheng Z, Wang W, Yuan Y, Hong Q, et al. Cigarette smoke-inactivated SIRT1 promotes autophagy-dependent senescence of alveolar epithelial type 2 cells to induce pulmonary fibrosis. Free Radical Biology and Medicine. 2021;166:116-27.
124. Zhao Y, Xu Y, Li Y, Xu W, Luo F, Wang B, et al. NF-kappaB-mediated inflammation leading to EMT via miR-200c is involved in cell transformation induced by cigarette smoke extract. Toxicological Sciences. 2013;135(2):265-76.
125. Zia S, Ndoye A, Nguyen VT, Grando SA. Nicotine enhances expression of the alpha 3, alpha 4, alpha 5, and alpha 7 nicotinic receptors modulating calcium metabolism and regulating adhesion and motility of respiratory epithelial cells. Research Communications in Molecular Pathology and Pharmacology. 1997;97(3):243-62.
