# Supplementary material for: Smoking and vaping alter genes related to mechanisms of SARS-CoV-2 susceptibility and severity: a systematic review and meta-analysis
Source: Eur Respir J. 2024 Jul 25;64(1):2400133. doi: 10.1183/13993003.00133-2024 (PMC11269771; doi:10.1183/13993003.00133-2024)
Supplement: Supplementary file 2 [file ERJ-00133-2024.Shareable.pdf]

SHAREABLE PDF

Smoking and vaping alter genes related to mechanisms of SARS-CoV-2 susceptibility and severity: a systematic review and meta-analysis

Rachel Bowsher[1,2], Timothy H. Marczylo[1], Karen Gooch[3], Alexis Bailey[2], Matthew D. Wright[1] and Emma L. Marczylo[1]

[1]Toxicology Department, UK Health Security Agency, Chilton, UK. [2]Pharmacology Section, St George's University of London, London, UK. [3]Vaccine Development and Evaluation Centre, UK Health Security Agency, Salisbury, UK.

Corresponding author: Rachel Bowsher (rachel.bowsher@ukhsa.gov.uk)

Check for updates

Shareable abstract (@ERSpublications)
Cigarette smoke and electronic cigarettes alter genes and subsequent pathways of interest involved in SARS-CoV-2 entry and hyperinflammatory immune responses. Direct implications on risk of infection and disease severity require more research. https://bit.ly/4eh060L

Cite this article as: Bowsher R, Marczylo TH, Gooch K, *et al*. Smoking and vaping alter genes related to mechanisms of SARS-CoV-2 susceptibility and severity: a systematic review and meta-analysis. *Eur Respir J* 2024; 64: 2400133 [DOI: 10.1183/13993003.00133-2024].

This extracted version can be shared freely online.

The content of this work is not subject to copyright. Design and branding are copyright ©ERS 2024.

This version is distributed under the terms of the Creative Commons Attribution Licence 4.0.

Received: 19 Jan 2024
Accepted: 23 May 2024

Abstract

Background Evidence for the impact of smoking on coronavirus disease 2019 (COVID-19) is contradictory, and there is little research on vaping. Here we provide greater clarity on mechanisms perturbed by tobacco cigarette, electronic cigarette and nicotine exposures that may impact the risks of infection and/or disease severity.

Methods Following Preferred Reporting Items for Systematic Reviews and Meta-Analyses (PRISMA) guidelines, the Ovid and Web of Science databases were searched. Study design and exposure-induced gene expression changes were extracted. Each study was quality assessed and higher confidence scores were assigned to genes consistently changed across multiple studies following the same exposure. These genes were used to explore pathways significantly altered following exposure.

Results 125 studies provided data on 480 genes altered by exposure to tobacco cigarettes, e-cigarettes, nicotine or severe acute respiratory syndrome coronavirus 2 (SARS-CoV-2). Genes involved in both SARS-CoV-2 viral-entry and inflammation were changed following exposure. Pathway analysis revealed that many of those genes with high confidence scores are involved in common cellular processes relating to hyperinflammatory immune responses.

Conclusion Exposure to tobacco cigarettes, e-cigarettes or nicotine may therefore impact initial host–pathogen interactions and disease severity. Smokers and vapers of e-cigarettes with nicotine could potentially be at increased risk of SARS-CoV-2 infection, associated cytokine storm, and acute respiratory distress syndrome. However, further research is required, particularly on e-cigarettes, to determine the biological mechanisms involved in perturbation of viral-entry genes and host–pathogen interactions and subsequent responses within the respiratory tract. This will improve our physiological understanding of the impact of smoking and vaping on COVID-19, informing public health advice and providing improved guidance for management of SARS-CoV-2 and other respiratory viruses.
